# Protein delivery to living cells by thermal stimulation for biophysical investigation

**DOI:** 10.1038/s41598-022-21103-9

**Published:** 2022-10-13

**Authors:** Francesco Torricella, Letizia Barbieri, Virginia Bazzurro, Alberto Diaspro, Lucia Banci

**Affiliations:** 1 Magnetic Resonance Center-CERM, University of Florence, via Luigi Sacconi 6, Sesto Fiorentino, 50019 Florence, Italy; 2 Interuniversity Consortium for Magnetic Resonance of Metallo Proteins (CIRMMP), via Luigi Sacconi 6, Sesto Fiorentino, 50019 Florence, Italy; 3 Department of Chemistry, University of Florence, via della Lastruccia 3, Sesto Fiorentino, 50019 Florence, Italy; 4 DIFILAB, Department of Physics, University of Genoa, via Dodecaneso 33, 16143 Genoa, Italy; 5 Nanoscopy, CHT Erzelli, Istituto Italiano di Tecnologia, Genoa, Italy

**Keywords:** Biochemistry, Biophysics, Developmental biology, Structural biology

## Abstract

Studying biomolecules in their native environment represents the ideal sample condition for structural biology investigations. Here we present a novel protocol which allows to delivery proteins into eukaryotic cells through a mild thermal stimulation. The data presented herein show the efficacy of this approach for delivering proteins in the intracellular environment of mammalian cells reaching a concentration range suitable for successfully applying biophysical methods, such as double electron electron resonance (DEER) measurements for characterising protein conformations.

## Introduction

During the last decades the possibility to characterize proteins, and biomolecules in general, in the intracellular environment of living cells opened up a new variety of experimental approaches called in-cell^1–3^. This approach focuses on the structural and functional investigation of biomolecules directly in their native environment^3,4^. In combination with the standard, in solution/in vitro structural biology approaches, driven by Nuclear magnetic resonance (NMR), together with Electron paramagnetic resonance (EPR) and Forster Resonance Energy Transfer (FRET), in-cell experiments provide a detailed description of the structural and functional properties of specific biomolecules in an almost physiological environment. The information obtained on globular and intrinsically disordered proteins (IDPs) by in-cell measurements, and in some cases, their differences with the in vitro description^5–8^, demonstrated the importance of their study in an environment as close as possible to the native one, where the crowding effects, specific and non-specific interactions, local pH changes, and redox conditions can affect the protein spatial organization^9–11^. The possibility to even look at protein–protein and protein-drugs interactions extended the applicability of in-cell experiments^12^.

One of the main bottlenecks for a wide spread application of in-cell experiments is represented by the sample preparation. Up to now the established methods for the preparation of in-cell NMR, EPR and FRET samples can be gathered in two main groups. In the first group, the investigated protein/is/are directly over-expressed inside cells using a DNA transformation^13^ (applied to bacteria/yeast) or infection/transfection^14^ (applied to insect and mammalian cells). The other group of methods involves the direct delivery of the desired protein from the resuspending buffer to the intracellular environment. In this second group, electroporation represents one of the main methods that can be used to deliver biomolecules in the intracellular environment^11^. In addition to it, the mechanical microinjection inside *Xenoupus laevis* oocytes has been extensively used^15,16^. Other methods, such as cell penetrating peptides, pore forming toxins, and hypo-osmotic shock have been shown to be alternative ways to deliver proteins into living cells^17–19^. Lastly, the use of nanoparticles^20^ and volume exchange for convective transfer^21^ (VECT) have been shown to be applicable to deliver proteins inside living cells. Interestingly, the use of silica nanoparticles has been demonstrated to be effective to deliver proteins and other biomolecules to a diverse set of mammalian cells^22^.

The delivery of proteins after their expression and purification represents the most common approach for EPR and FRET measurements which generally require a Site Directed Labelling (SDL) with paramagnetic or fluorescent probes. In recent years, with the expansion of the *E. coli* genetic code introduced by Shultz et al.^23^, some works showed how the in vivo labelling of proteins over-expressed in *E. coli* could represent a valid alternative for the preparation of in-cell samples for EPR spectroscopy^24^. However, the necessity of two orthogonal plasmids, for the expression of the protein of interest and of the relative tRNA and aminoacyl-tRNA synthetase for the desired unnatural amino acid, makes this approach demanding for a wide use in structural biology studies.

For in vitro EPR-based measurements, nitroxide labels are extensively used^25,26^. As the common, commercially available 3-maleimido-Proxyl (ma-Px) radical is sensitive to bioreduction in the cell, the standard, slow delivery methods (e.g.: the one that requires cell recovery) are not suitable for in-cell application^27^. In the last decades, the introduction of shielded nitroxides, trityl radical, and gadolinium tags somehow overcame the problem of paramagnetic stability of non-shielded nitroxide labels^28–30^. However, faster and simpler methods for tagged protein insertion in the cells, are still required which can significantly contribute to make these measurements more effective.

The present work reports on the application to mammalian cells of a protocol for protein delivery already developed for bacteria and yeast^31^. It is based on the use of heat shock (HS) treatments which allows to reach delivered protein concentrations suitable for biophysical investigations. Recently, it has been shown that Double Electron Electron Resonance spectroscopy (DEER) either in vitro or in-cell can be successfully performed at the nanomolar^32,33^ concentration range, which is the level of concentrations reached with this proposed method. The data here reported show that the HS delivery developed by us allows to effectively employ commercial nitroxide tags such as ma-Px, due to the fast protocol and the relatively short death time for sample preparation (~ 15 min). We have optimized the HS delivery method for three different human cell lines using Ubiquitin (Ub) and the Green Fluorescent Protein (GFP) as model proteins.

## Results and discussions

**Figure 1:**
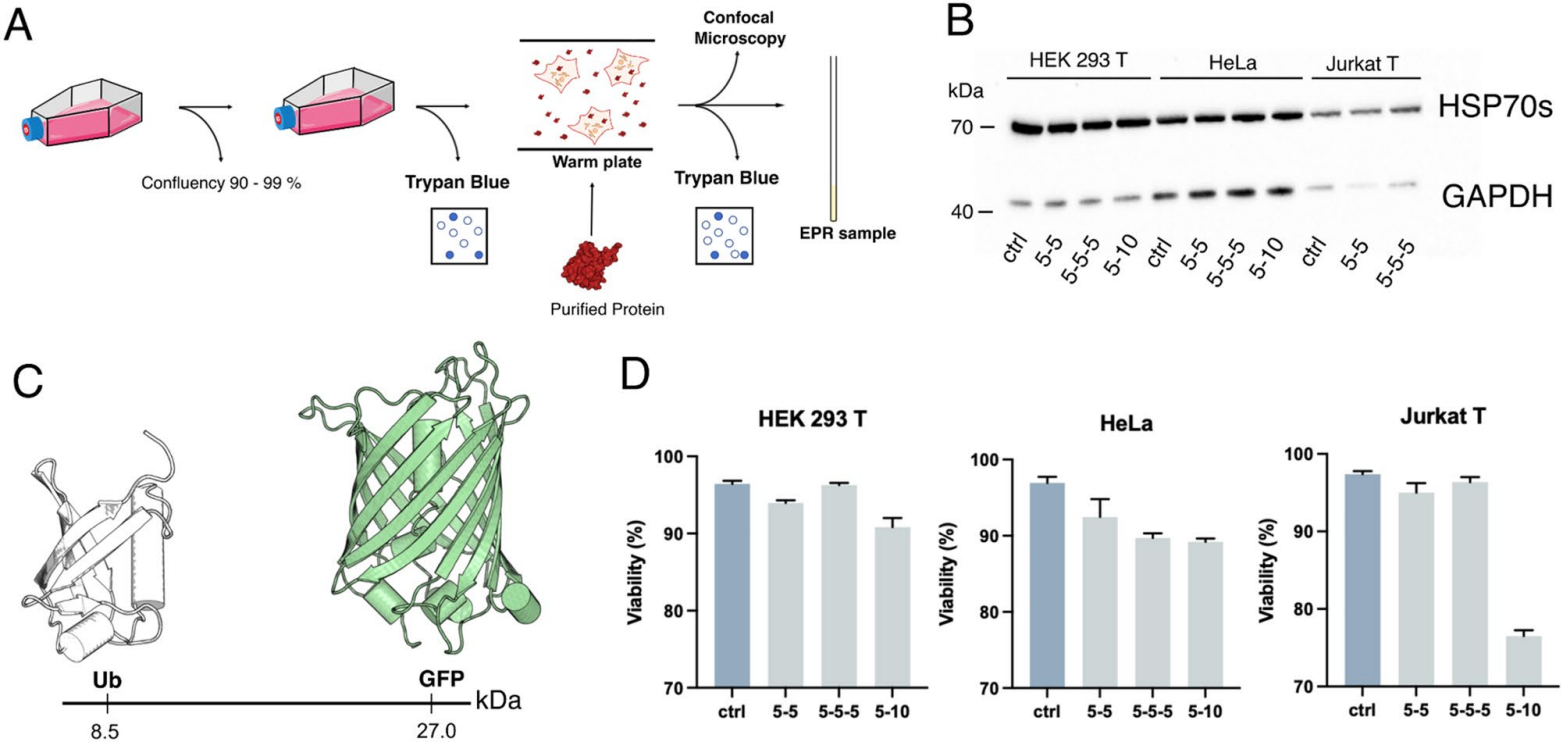
(**A**) Schematic representation of the developed thermal stimulation delivery method (HS). (**B**) HSP70s WB assay conducted on the HEK 293 T, HeLa and Jurkat T cells after the thermal stimulation delivery. (**C**) PDB structures of the proteins used in this work (Ub: 1UBQ.pdb GFP: 1GFL.pdb). (**D**) Trypan blue Viability assay for control and treated cells with the three tested thermal stimulation schemes in absence of external protein. The raw western blot membrane is shown if the Supplementary Information, Fig. S3.

**Figure 2:**
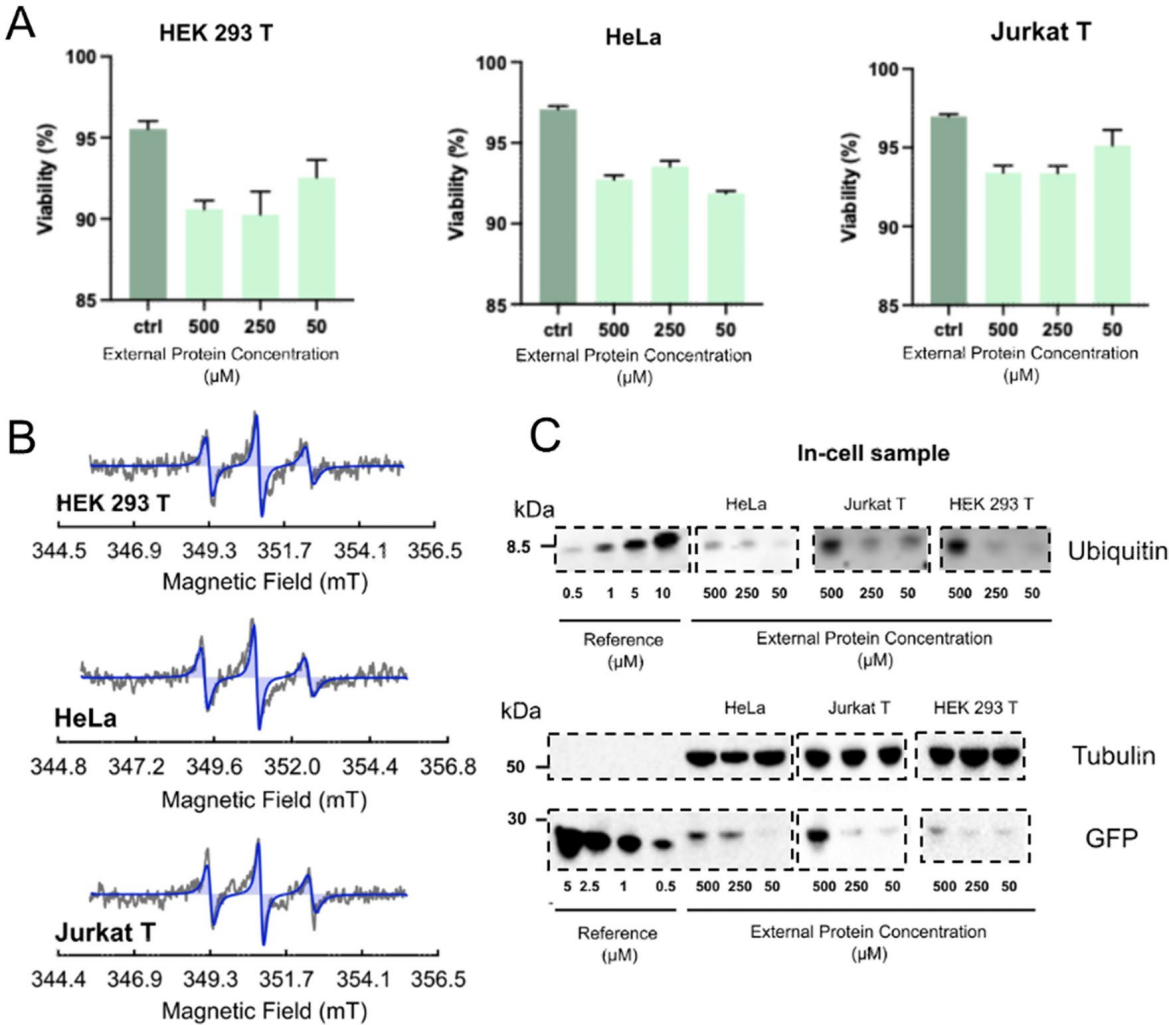
(**A**) Viability assay conducted using the 5-5 HS delivery setup in presence of variable external Ubiquitin protein concentrations. (**B**) grey-cw-EPR spectra acquired at RT on HeLa, HEK 293 T and Jurkat T cells where ubiquitin A28C ma-Px-spin labelled was delivered using the 5-5 delivery scheme and an external protein concentration of 500 μM. Blue-cw-EPR spectra acquired for an in vitro reference sample of ubiquitin A28C ma-Px-spin labelled protein at 100 μM. (**C**) In-gel fluorescence PAGE and Western blot assay conducted on Ub A28C-atto488 and GFP delivered cells using the 5-5 delivery setup. In each assay references at known concentrations were used (Supplementary Information, Experimental Methods, Figs. S1–S3). All gels reported in this figure have been selected from the raw gels reported in the Supplementary Information.

Despite some of the effects on cells due to mild hyperthermia have been investigated in literature^34^, thermal treatment has never been used for in-cell sample preparation for spectroscopic studies applied to human cells. To obtain a physiological-like in-cell sample, we implemented a fast, simple and effective protocol for delivering proteins inside mammalian cells, based on multiple thermal stimulations, which we already developed for bacterial and yeast cells^31^. We validated the protocol on HEK 293 T, HeLa, and Jurkat T human cell lines. As reported in Fig. 1 we tested three different HS delivery schemes, where the time cycles at which cells were kept at 42 °C were varied by different intervals (5 and/or 10 min) (Supplementary Information, Experimental Methods). Cell viability, tested with Trypan blue, resulted to be in the range of 89–95% after the delivery protocol either in absence (Fig. 1) or in presence of different external protein concentrations (500 μM, 250 μM and 50 μM) (Fig. 2, Supplementary Information, Experimental Methods) and in absence of cell recovery.

First, the validity of the method and the absence of any induced perturbation on the cells was assessed. To this purpose, the HSP70s cellular content after the HS cycles was estimated with respect to the control protein expression level. Western Blot (WB) analysis (Fig. 1) showed essentially the same expression level in control cells vs HS treated cells indicating that the heat treatment on the cells does not produce any cellular shock which would have altered the expression levels of HSP70s.

**Figure 3:**
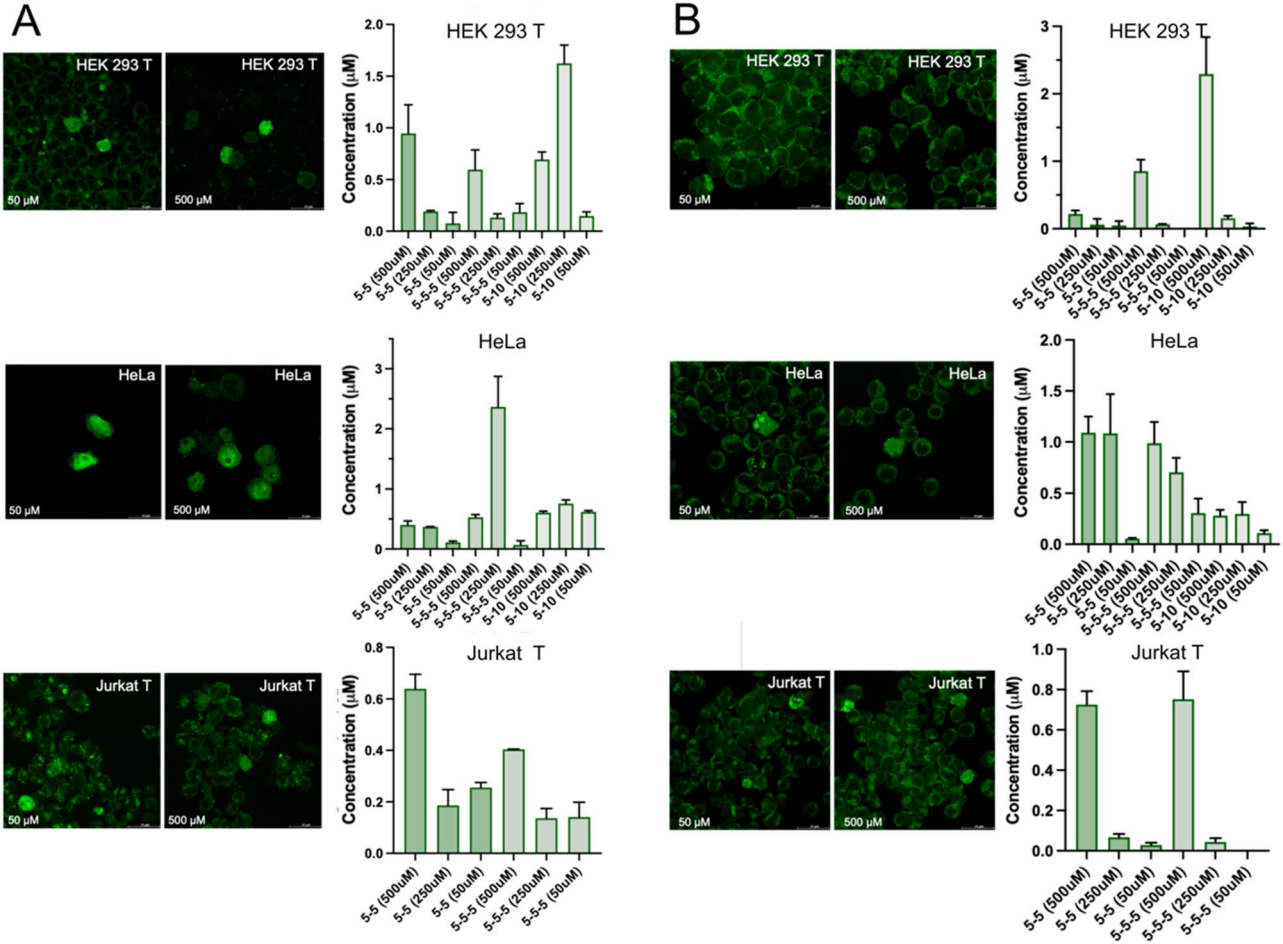
(**A**-left) Confocal images acquired on the delivered cells with Ubiquitin-A28C-atto488 protein. Column A was acquired on cell samples delivered with 50 μM external protein concentration, while the cells in the column B were delivered with 500 μM external concentration using the HS 5-5 delivery set-up. (A-right) In-gel fluorescence PAGE derived bulk intracellular concentration respectively for the Ubiquitin-atto488 under different HS delivery set-up (Supplementary Information, Figs. S1–S3). (**B**-left) Confocal images acquired on GFP delivered cells delivered with the HS 5-5 delivery set-up. Cells in column C were delivered with 50 μM external concentration while those in column D were treated with an external concentration of 500 μM. (**B**-right) Western Blot derived bulk intracellular concentration respectively for the GFP delivered cells under different HS delivery set-up (Supplementary Information, Figs. S1–S3).

The efficacy of the HS delivery was then tested for GFP and Ub through WB and in-gel fluorescence PAGE analysis on the lysate of cells to which either GFP or Ub, tagged with the maleimide atto-488 fluorophore (Ub-A28C-atto-488), were delivered (Supplementary Information, Experimental Methods, Figs. S1–S3). This analysis estimated the bulk concentration of the internalized protein to be in the range of 0.1–3.5 µM for the various external protein concentrations (Fig. 3 and Supplementary Information, Figs. S1–S3). High-resolution confocal fluorescence microscopy (Fig. 3) and Z-stack reconstruction (Supplementary Information, Experimental Methods, Fig. S4) confirmed the protocol effectiveness towards protein delivery, by assessing that the vast majority of the delivered proteins is inside the cell, and showed that the protocol essentially does not affect the physiological intactness of the treated cells (Fig. 3).

A series of EPR spectra were then performed to show the potentialities of this simple and effective protocol for protein in-cell insertion. X-band cw-EPR spectra at room temperature were acquired on cells treated with Ub A28C spin labelled with ma-Px at two different external protein concentrations (Supplementary Information, Figs. S5, S6). Using the 5-5 HS delivery scheme, we made the overall lag time before the start of the EPR experiments very short, thus obtaining the maximum signal intensity of the ma-Px radical with respect to its bioreduction process. From the double integral of the EPR signals, we derived an overall protein-spin bulk concentration in the range of 0.30–0.60 μM for the delivered Ub A28C-ma-Px cells (Fig. 2). To assure that the signal is coming from the intracellular delivered protein, the cells were washed 5 times with fresh PBS. Afterwards, for each wash sample the X-band RT cw-EPR spectra have been acquired (Supplementary Information, Fig. S8). Additionally, the EPR cellular background at RT was acquired to validate the complete absence of signals in the X-band cw-EPR nitroxide transition window.

**Figure 4:**
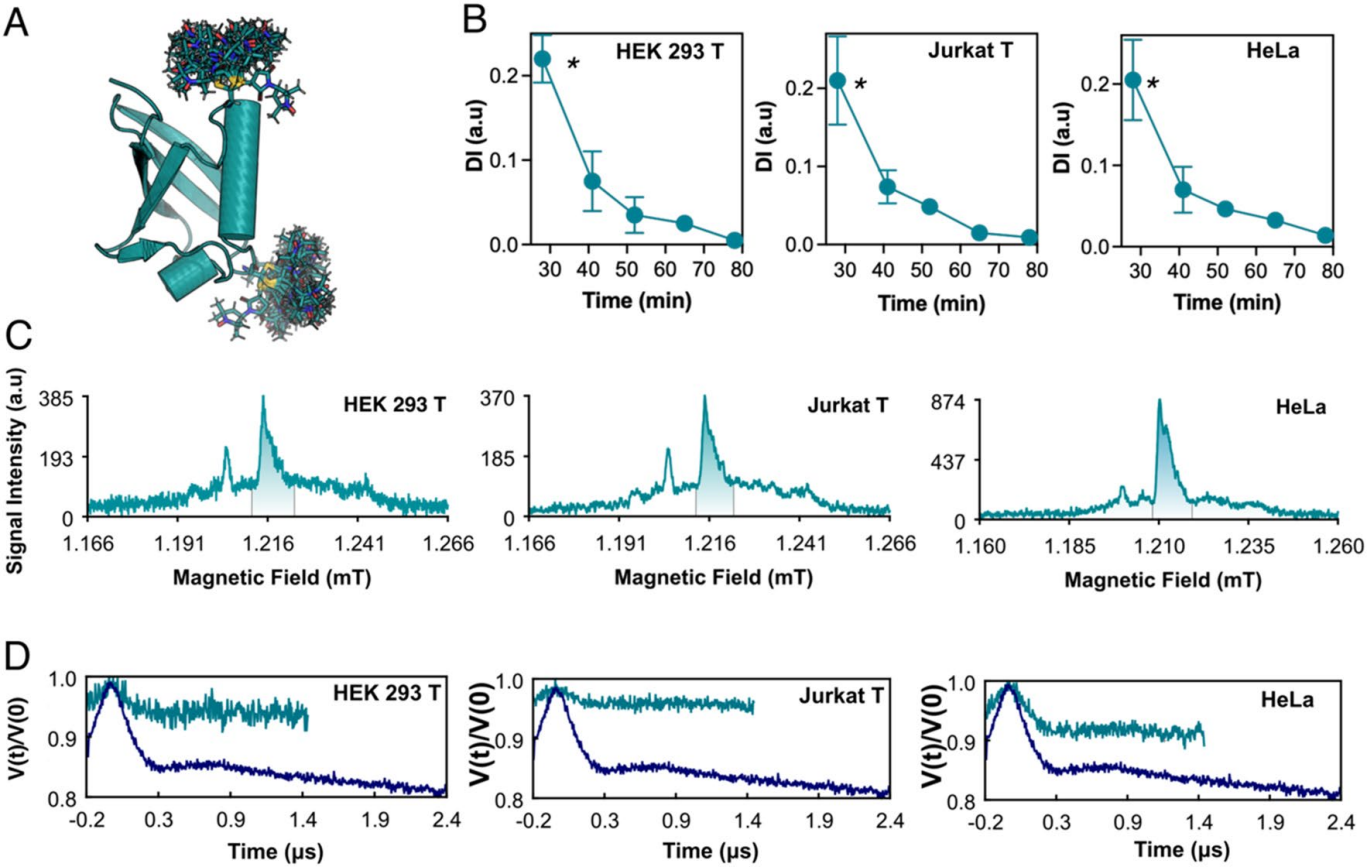
(**A**) Model structure of human ubiquitin protein labelled with Maleimide DOTA Gadolinium tag in the S20C and G35C position derived from MMM^44^. (**B**) Nitroxide double integral signal decay over time extracted from the RT cw-EPR X-band spectra for the Ubiquitin S20C/G35C ma-Px delivered cells. The spectra DI (Double Integral) were plotted against the overall time course. The first point time point take into account the death time before the sample acquisition composed by the delivery and preparation time. Each cw-EPR experiment was set to be ~ 13 min in acquisition time. The asterisks represent the relative time position in which the pulsed EPR samples were frozen. (**C**) Acquired EDFS spectra for the delivered cells, recorded at 50 K at the center of the resonator dip under overcoupling conditions. The cellular Manganese (Mn^2+^) EDFS signal is contributing to the spectra with the broad transition and the downfield peak in respect to the nitroxide signal. (**D**) DEER traces of the in vitro (blue trace) and in-cell samples (cyan traces) acquired at 50 K.

**Figure 5:**
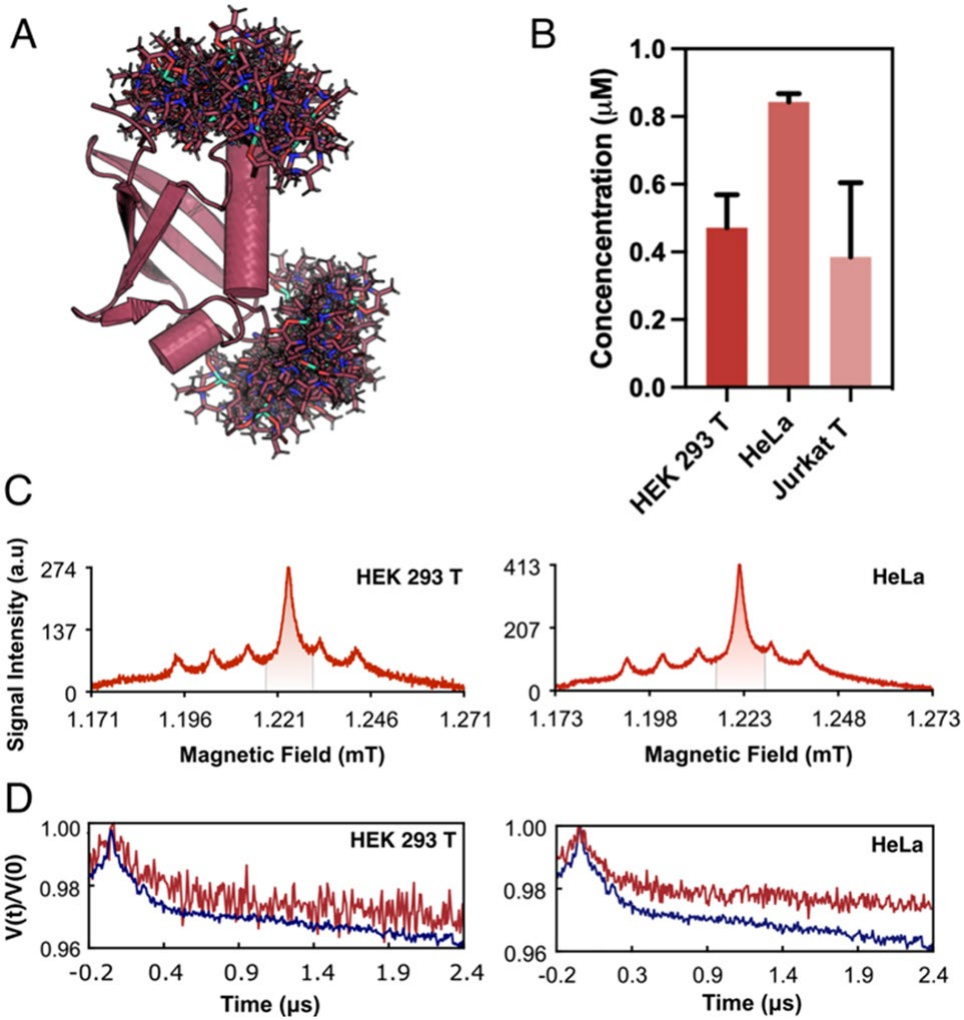
(**A**) Model structure of human ubiquitin protein labelled with doubly Maleimide DOTA Gadolinium tag in the S20C and G35C position. (**B**) Derived bulk intracellular concentration for the Ub-ma-DOTA-Gd^3+^ delivered cells. The concentration was estimated interpolating the Integral value of the in-cell spectra Gd^3+^ transition with a calibration curve. (**C**) Acquired EDFS spectra for the HEK 293 T and HeLa cells over the center of the resonator dip under overcoupling conditions. (**D**) DEER spectra acquired for the in vitro (blue traces) and the relative in-cell sample (red traces).

Our protocol also allowed the collection of more sophisticated experiments such as DEER. For this purpose, the Ub S20C/G35C double mutant was doubly labelled with either two ma-Px radicals or two maleimide-DOTA-Gd^3+^ (ma-DOTA-Gd^3+^) spin labels and then delivered inside each of the three used cell lines with the 5-5 HS delivery scheme (Supplementary Information, Experimental Methods). Based on the double integral of the double nitroxide labelled protein cw-EPR signals, we estimated an overall protein-spin concentration in the range of 0.28–0.70 μM (Supplementary Information, Fig. S9). cw-EPR spectra recorded over time (Supplementary Information, Experimental Methods) after the ma-Px doubly labelled Ub S20C/G35C protein insertion showed the complete disappearance of the nitroxide radical signal after 40 min from the end of the sample preparation (Fig. 4). This measurement time is however enough to obtain dynamical information from the cw-EPR spectra (Fig. 2). Additionally, Q-band echo detected field sweep (EDFS) conducted on the cells with the same doubly nitroxide tagged Ub showed the characteristic signal arising from nitroxide moiety (Fig. 3 and Supplementary Information, Fig. S10). Indeed, the analysis of in-cell DEER traces (Supplementary Information, Fig. S12, and Supplementary Table 1 for spectra set-up) gave a view on the distance distribution for the doubly labelled delivered protein even in presence of a decreasing in the modulation depth of the nitroxide DEER traces (Fig. 5), due to the partial reduction of nitroxide inside the cell^35^.

EDFS and DEER experiments were successfully performed also on two of the three used cells lines where Ub S20C/G35C doubly labelled with ma-DOTA-Gd^3+^ was delivered (Fig. 5). For all delivered cell lines, the bulk intracellular concentration for the Ub-ma-DOTA-Gd^3+^ was estimated interpolating the Integral value of the in-cell spectra Gd^3+^ transition with a calibration curve. The Ma-DOTA-Gd^3+^ label represents an optimal, commercially available biocompatible spin label as previously shown^19,32^. The stability of this radical in the intracellular reducing environment allowed the detection of the signal on cells delivered with 50 μM external Ub- ma-DOTA-Gd^3+^ concentration.

Overall, the acquired DEER spectra for both Ub-ma-Px and Ub-ma-DOTA-Gd^3+^ were of reasonable quality so that the distance distribution from the DEER can be estimated, through DeerLab^36^, DD^37^ and WavSVD^38^ approaches (Supplementary Information, Experimental Methods, Figs. S11–S13).

## Conclusion

Here we present an easy, fast, and wide applicable method for delivering proteins inside mammalian cells. This protocol gives the possibility to investigate biomolecules inside their natural environment, reaching bulk concentrations in the sub micromolar range. The HS delivery method here reported does not impact the cell viability and therefore it can be performed even in absence of cell recovery. We additionally showed how the use of a cell recovery of almost 1 h does not change the overall viability of the cell and the derived EPR signal of the delivered doubly Ma-DOTA-Gd^3+^ labelled ubiquitin (Supplementary Information, Fig. S14). Moreover, the heat cycle exposure at 42 °C does not alter the physiological processes of the cells, as indicated by the unaltered expression levels of HSP70s proteins, which are reporters of the biological alterations induced by heat shock. To assess the efficacy of our method, confocal microscopy 2D and 3D images showed the effective delivery of GFP and Ub inside the cells. The limited preparation dead time (~ 12–15 min) of our HS delivery protocol allows the use of commercially available spin labels, such as ma-Px, which usually suffer from bioreduction and extends the applicability of the stable ones (e.g.: shielded nitroxide, trityl radical). cw-EPR experiments, as expected, on spin labelled Ub allowed to assess some minor changes in its dynamics between the in vitro and the in-cell localization. DEER experiments could be successfully acquired on proteins inside the cells which gave the possibility to extract distance distribution between the two labelling sites of Ub S20C/G35C. In this regard, we have shown the powerful combination of the heat shock delivery with the use of Ma-proxyl and the Ma-DOTA-Gd^3+^. In both cases, we found that the best in cell sample preparation set-up for PDS EPR is the use of 5-5 HS delivery scheme with 500 μM external protein concentration for all used cell lines. The Ma-DOTA-Gd^3+^ represents a valuable bioresistant paramagnetic label which however suffers of an increased flexibility compared to nitroxide. This increased flexibility can be appreciated from the broader distance distribution obtained both in vitro and in cell (Supplementary Information, Figs. S11-13). Indeed, as previously shown by Goldfab et al. an increased signal-to-noise ratio and a narrow distance distribution can be obtained using different gadolinium-based labels^39^ (e.g.: DO3MA-Gd and DO3A-Gd).

As final comment, we would like to add that, while we have here shown the potentialities of this protein-cell delivery approach, there is still a wide room for improvements in terms of applicability and optimization such as the combination with other spectroscopic methods.

## Methods

### Western Blot and in gel fluorescence assay

#### Ubiquitin

Human ubiquitin protein containing either the A28C or S20C/G35C mutations was expressed using a pET-21a. The expression followed the protocol reported in the previous work Torricella et al.^31^. Briefly *E. coli* BL21(DE3) strain was transformed with the above mentioned plasmid and, they were grown to mid-log phase at 37 °C in LB (Luria–Bertani) medium. At 0.6 OD_600_ value, the cells were inducted with 1 mM of isopropyl β-d-1-thiogalactopyranoside (IPTG). Right after the induction cells were grown for other 5 h at 37 °C under gentle shaking. Indeed, the cells were harvested ad resuspended in the relative lysis buffer (50 mM Tris–HCl, 1 mM EDTA, 0.05% Triton X-100, pH 7.6) and lysed via sonication. The sonication was conducted using 3 s ON and 9 s OFF at 60% of amplitude for 40 min in a ice.

#### GFP

A pET-28a vector encoding for GFP protein was used to transform *E. coli* BL21 (DE3) (Stratagene) cells and the culture was grown at 37 °C in LB. At mid-log phase the expression (OD_600_: 0.6 A) was induced with 1 mM of IPTG and the culture was grown for other 5 h. The cells were then harvested and resuspended in lysis buffer (10 mM Tris, 100 mM NaCl, 50 mM NaH_2_PO_4_, 5 mM imidazole, pH 8) and lysed by sonication (3 s ON, 9 s OFF, at 60% of amplitude for 40 min). After clarification, the lysate was loaded onto a 5 mL HisTrap™ FF affinity column (Cytiva) and eluted with a linear gradient of elution buffer (10 mM Tris, 100 mM NaCl, 50 mM NaH_2_PO_4_, 500 mM imidazole, pH 8). Finally, the protein buffer was exchanged with PBS pH 7.4 (Gibco).

### Protein site directed spin labeling and purification

#### Single and double nitroxide spin labelling procedure

Purified Ubiquitin either carrying the A28C or S20C/G35C mutations were initially incubated in a ratio 1:10 with 1,4-Dithiothreitol (DTT) to reduce the available cysteines residues. Afterwards, each protein solution was incubated with 10 equivalents of 3-maleimido-Proxyl (Sigma Aldrich), for each available cysteine. The labelling reaction was incubated overnight at 4 °C to maximize the labelling yield. After the incubation time each reaction pot was purified using a PD-10 column (Cytiva) collecting fractions of 200 μL. The spin labelled protein was then separated from the unreacted spin label looking at the cw-EPR X-band RT spectra.

#### Double gadolinium spin labelling procedure

As for the double nitroxide Ubiquitin preparation, the purified S20C/G35C protein was initially incubated with 1:10 molar ratio of DTT to reduce the two available cysteines. Afterwards at the protein solution 5 equivalents of maleimide-Gd-DOTA were added. The reaction was kept at 4 °C for an Overnight incubation and afterwards the spin labelled protein was purified using an HiLoad 16/600 Superdex 75 pg column (GE Healthcare) exchanging the buffer with the delivery buffer constituted by PBS (gibco) and removing the unreacted Gd^3+^.

### Human cell cultures

HEK 293 T cells (ATCC CRL-3216) and HeLa cells (obtained from Swiss Institute for Experimental Cancer Research) were maintained in Dulbecco’s Modified Eagle Medium (DMEM) high glucose (Gibco) supplemented with l-glutamine, antibiotics (penicillin and streptomycin) and 10% fetal bovine serum (FBS; Gibco) in uncoated 75 cm2 plastic flasks. Jurkat cells (purchased from the InterLab Cell Line Collection bank (ICLC HTL01002)) were maintained in RPMI 1640 medium (Gibco) supplemented with MEM Non-Essential Amino Acids (NEAA; Gibco), sodium pyruvate (Gibco), antibiotics (penicillin and streptomycin) and 10% fetal bovine serum (FBS; Gibco) in uncoated 75 cm^2^ plastic flasks. All cell lines were grown at 37 °C and 5% CO2 in a humidified atmosphere.

### Delivery protocol

#### Cell preparation

Each used human cell lines were initially detached (HEK 293 T) or harvested (Jurkat T) from their own growing media at 90%-99% confluency. HEK 293 T and HeLa cells, were collected using the same procedure. Briefly, cells were washed twice with phosphate-buffered saline (PBS, gibco) and subsequently detached with trypsin–EDTA (0.05% (wt/vol). Afterwards, DMEM + 10% FBS was added to inactivate trypsin. The cell suspension was centrifuged at 800 g for 5 min at room temperature, collecting the cell pellet. The cell pellet was washed with PBS buffer to remove the residual medium. Concluding, the cell pellet was finally collected by centrifugation at 800 g for 5 min at room temperature. On the other hand, Jurkat cells were resuspended in RPMI 1640 medium to break up any clumps of cells and centrifuged at 800 g for 5 min at room temperature. The collected cell pellet was washed with PBS and centrifugated again at 800 g for 5 min at room temperature.

#### Heath shock delivery protocols

The needed amount of cell was then used for the delivery protocol made by different cycle of heat (42 °C) and cold (ICE) temperature incubation. The cells (In the range of 3 × 10^6^ cells for confocal based experiments and 6 × 10^6^ cells for EPR based measurements) were resuspended in 50 mL of PBS buffer and 50 mL of PBS solution containing different concentration (100 μM, 500 μM and 1000 μM) of the interested protein to be internalized. Afterwards, the proper delivery method was performed in three different ways. In the first case the 5-5 set-up is composed by two 5 min incubations at 42 °C, interspersed by a 2 min incubation in ICE (defined by the symbol -). Subsequently to the last incubation at 42 °C the cells were additionally incubated in ICE for 2 min and then the cell pellet was collected by centrifugation at 800 g for 2 min. The cell pellet was the washed five times with 200 mL of fresh PBS to remove all of the protein not delivered (Fig. S8), collecting the cells each time by centrifugation as before. Each fraction was checked by WB, in gel fluorescence and cw-EPR. Indeed, we tested other two thermal delivery set-up which are presented as 5-5-5 and 5-10. This last set-up differs from the 5-5 by the number of heat/cold incubations. The 5–5-5 is composed by three 5 min incubations at 42 °C and two 2 min incubations in ICE. On the other hand, the 5-10 is composed by one 5 min incubation at 42 °C, one 2 min incubation in ICE and a concluding 10 min incubation at 42 °C. Indeed, the methods follow the same steps after the last incubation at 42 °C. For in cell RT X-band EPR, the cells after the last wash were resuspended in 20 mL of PBS placed into a quartz tube (O.D 1.6 mm) and measured inside the resonator. The sample for pulsed EPR were resuspended in 20 mL of PBS and instantly frozen after the last wash in a quartz tube (O.D 1.6 mm). Concluding, the confocal miscopy sample were prepared as described in the following section.

### Trypan blue vitality test

The impact of the delivery procedures on cells viability were assessed using Trypan blue exclusion test on the resuspended pallet right after the last wash. After the last wash the cells were harvested by centrifugation (800 g, 3.0 min, RT) and resuspended in 180 mL of DMEM. Subsequently 10 mL of this suspension were mixed with 10 mL of 0.4% Trypan Blue. All measurements were acquired in triplicate with the automated LUNA-II cell counter (Logos Biosystem, Inc.). This method has been used either in presence or absence of external protein concentration.

### Western Blot (WB) assay on HSP70s

GFP concentrations in all cell lines lysates were determined by western blot analysis by using samples of purified protein at increasing dilutions as references. Delivered and control cells were always lysate in 150 mL. GFP was stained with a rabbit polyclonal anti-GFP antibody (Antibodies.com: A252, diluted at 1:2000). HSP70 was stained with a rabbit monoclonal anti-HSP70 antibody (Antibodies.com: A13125, diluted at 1:1000). To normalize the number of cells of different samples, western blots were performed against the cytoplasmic marker GAPDH, using a rabbit polyclonal anti-GAPDH antibody (Abcam: ab181603, diluted at 1:10,000) or against beta-Tubulin, using a rabbit polyclonal anti-beta tubulin antibody (Abcam: ab6046, diluted at 1:1000). Goat anti-rabbit IgG (whole molecule)-peroxidase secondary antibody (Sigma: A0545) was used for detection, diluted at 1:80,000. For detection, a LiteAblot EXTEND chemiluminescent substrate (EuroClone) was used. Densitometry analysis was performed with ImageJ^40^.

### In gel fluorescence on ubiquitin-A28C-atto-488

Briefly the in gel-fluorescence PAGE was conducted on the lysate of the cell delivered with the Ubiquitin A28C labelled with the atto-488. Delivered and control cells were always lysate in 150 mL. The samples for the PAGE were resuspended with the loading dye solution free from DTT. Subsequently Mini-PROTEAN TGX gels (BIORAD) were used as electrophoresis matrix. The band intensities were acquired irradiating the gel at the maximum wavelength absorption of the atto-488 fluorophore. Densitometry analysis was performed with ImageJ^40^ using a calibration curve made by standard sample of ubiquitin atto-488 at known concentration. Each measurement was conducted in duplicate.

### X-band room-temperature cw-EPR spectroscopy of in vitro/in cell sample

All cw-EPR experiments were acquired using a Bruker ELEXYS E580 spectrometer equipped with a HIGH Q ER4122SHQE operating at X-band using EPR quartz tube with 1.6 mm O.D and 1 mm I.D. The spectroscopic setting for the in vitro measurements was kept as following: ν = 9.874 GHz; center field = 3500 G; sweep width = 150 G; microwave power = 126 mW; modulation frequency = 100 kHz; modulation amplitude = 1 G; conversion time = 25 ms; sweep time = 25.6 s; scans = 25).

All in cell samples were recorded in a time resolved set-up with the same conditions except that a time axis was defined to follow the entire reduction of the spin-label inside the intracellular environments. Each experiment was acquired in 13 min.

The start of the measurements was in average 12–14 min after the last thermal treatment, and the zero time was set as the starting time of the delivery protocol.

### Q-band echo-detected field-sweep of in vitro/in cell sample

in vitro and in cell samples Echo-detected field sweep (EDFS-EPR) experiments were acquired observing the amplitude variations of the spin echo made by the following sequence: π/2 – τ – π – τ - *echo*. All the experiments were acquired using a Bruker ELEXYS E580 X/Q-band spectrometer equipped with a pulse 10 W Amp Q amplifier, an EN 5107D2 Q-band EPR/ENDOR probe-head and a continuous He-flow cryostat (Oxford Instruments) coupled with a temperature controller (Oxford Instruments). The temperature was kept at 50 K for the nitroxide based measurements and 10 K for the ones using Gadolinium as paramagnetic probe. The EDFS were acquired both at the pump and detection frequency optimizing the pulses length (t_p_) using the mw-nutation sequence: t_p_ - τ - π/2 -τ - π - τ- det. Additionally the EDFS spectra acquired on Gd delivered samples (Fig. S9) were acquired using the same spectroscopical setup. The pulses were optimized as described previously and the video gain (VG) was kept the same for the 250 μM and 500 μM concentration, and increased for the 50 μM external concentration. To probe the reproducibility of the here proposed delivery method, we acquired in duplicate the EDFS of different delivered cells.

### Q-band DEER spectroscopy in vitro/in cell sample

All Q-band DEER/PELDOR measurements were performed at the cryogenic temperature of 50 K or 10 K under over-coupling conditions. All experiments were conducted with the same spectrometer set-up as before. In vitro and in cell samples (~ 20 mL) with 20% v/v of glycerol were inserted in quartz capillary tubes with an O.D of 1.6 mm and were instantly frozen in liquid nitrogen. Specifically, in the case of in cell samples, the cells were washed 5 times with fresh PBS. Afterwards the cells were resuspended in PBS containing the above mentioned 20% v/v glycerol.

All DEER experiments on both in vitro and in cell sample were acquired using the standard 4-pulse sequence^41^, employing rectangular pulses for Nitroxide based measurements and Gaussian pulses for the Gadolinium based samples. All the pulses either rectangular or gaussian were optimized both at the detection and pump frequency using the above reported mw-nutation sequence. For both Nitroxide DEER and Gadolinium DEER, the ν_pump_ was set at − 70 MHz offset from the ν_detection_. For the in vitro experiments the πdetection was measured to be equal to 70 ns and the relative πpump was set to 34 ns. The pump pulse at the ν_pump_ was applied 60 ns before the second pulse of the detection sequence and moved with 4 ns time-step for Nitroxide DEER and with 8 ns time-step for the Gadolinium DEER. 50 shots per points were acquired with the optimized SRT. The data analysis was performed using three different routines. In detail, Deerlab^36^ DD^37^ and WavSVD were implemented to retrieve the distance distributions from the acquired DEER traces. DeerLab suite was implemented using the available script and optimizing the regularization parameter using either the AIC or GCV criterion. Additionally for each calculated distance distribution a bootraspped uncertainty method with 1000 bootstrap sample have been used. On the other hand, DD MATLAB suite were implemented using a unimodal Gaussian model in combination with the built-in background exponential decay function. Concluding the WavSVD^42,43^ which firstly uses a wavelet approach to filter out the noise from the experimental trace and secondly gives the possibility to extract the distance distributions from by singular value decomposition (SVD). For all denoising Daubechis 6 wavelet group was used. Consequently, the denoised trace were used for SVD reconstruction employing the Picard Plot^38^ to estimate the value of SVD.

### Confocal microscopy

All Confocal microscopy sample were prepared as follow. The treated cells were fixed using para-formaldehyde (4% in PBS) for 15 min and then washed twice with fresh PBS. Right after the cells were placed on poly-lysine treated coverslips and mounted on the slide with ProLong Gold Antifade mountant (Thermo Fisher) .Fluorescence measurements were performed with a Stellaris8 Falcon TauSTED (Leica Microsystems, Mannheim, Germany) operating as inverted microscope. A white light laser provided the desired excitation wavelength setting a notch filter at 488 nm, and the samples were viewed through a plan-apochromatic oil immersion objective × 100/1.40 NA. 2D images (1024 × 1024 × 16 bit) were obtained by scanning with a speed of 200 Hz. An avalanche photodiode was used as detector in the 500–650 nm range. 3D acquisitions were made with a Z-step size of 0.18 µm. The pinhole was set at 1.0 Airy size for all the measurements.

## Supplementary Information

Supplementary Information.

## Data Availability

All data generated or analysed during this study are included in this published article. Any additional datasets are available from the corresponding author on reasonable request.
