# Supplementary material for: Protein delivery to living cells by thermal stimulation for biophysical investigation
Source: Sci Rep. 2022 Oct 13;12:17190. doi: 10.1038/s41598-022-21103-9 (PMC9561116; doi:10.1038/s41598-022-21103-9)
Supplement: Supplementary file 1 — Supplementary Information. [file 41598_2022_21103_MOESM1_ESM.docx]

Supplementary Information

**Protein Delivery to Living Cells by Thermal Stimulation**

**for Biophysical Investigation**

Francesco Torricella, Letizia Barbieri, Virginia Bazzurro, Alberto Diaspro and Lucia Banci

Table of Contents

[1. Supplementary Figures and Tables 2](#_Toc110591324)

[Western Blot and in gel Fluorescence assay 2](#_Toc110591325)

[Ub-A28C and Ub-S20C/G35C ma-Px X-band cw-EPR in vitro spectra 5](#_Toc110591326)

[X-band Ub-A28C and Ub-S20C/G35C ma-Px delivery test 6](#_Toc110591327)

[In vitro Q-band EDFS Ub-S20C/G35C ma-Px 6](#_Toc110591328)

[X-band cw-EPR in cell control experiments 7](#_Toc110591329)

[Q-band EDFS Ub-S20C/G35C maleimide-Gd-DOTA delivery test 8](#_Toc110591330)

[In cell Q-band EDFS Ub-S20C/G35C ma-Px 9](#_Toc110591331)

[In vitro Nitroxide Gadolinium DEER/PELDOR set-up and data analysis 10](#_Toc110591333)

[In cell Nitroxide DEER/PELDOR set-up and data analysis 11](#_Toc110591334)

[In cell Gadolinium DEER/PELDOR set-up and data analysis 12](#_Toc110591335)

[In cell Q-band EDFS Ub-S20C/G35C Ma-DOTA-Gd^3+^. 13](#_Toc110591337)

[In cell Q-band Echo Decay curves 13](#_Toc110591338)

[Table. 1: Parameters used for the in vitro and in cell DEER experiments on the doubly labelled nitroxide and gadolinium Ubiquitin based samples 14](#_Toc110591340)

[Table. 2: Nitroxide doubly labelled Ub S20C/G35C in-cell/in vitro derived distances 14](#_Toc110591341)

[Table. 3: Gadolinium doubly labelled Ub S20C/G35C in-cell/in vitro derived distances 14](#_Toc110591342)

# **Supplementary Figures and Tables**

# **Western Blot and in gel Fluorescence assay**


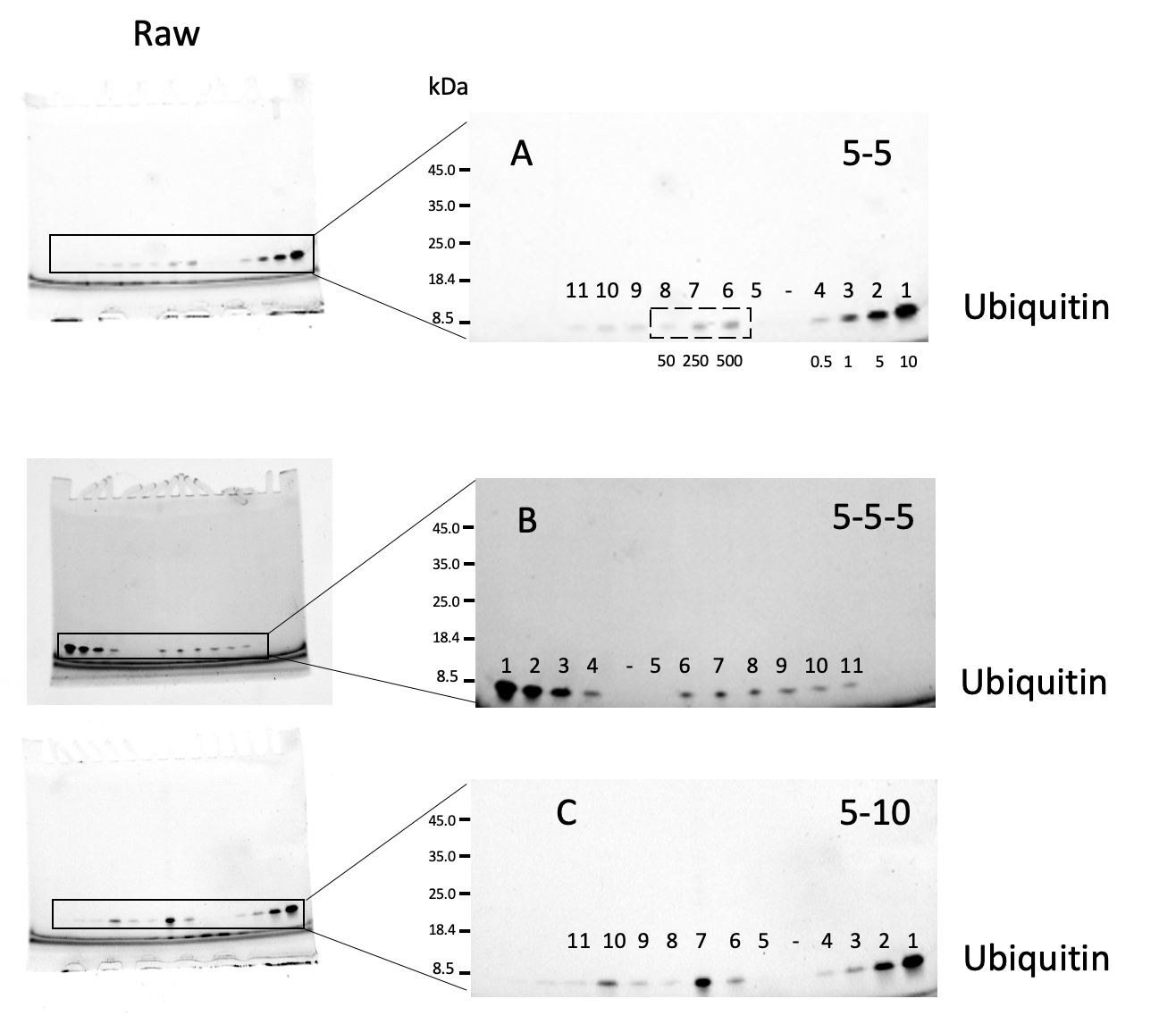


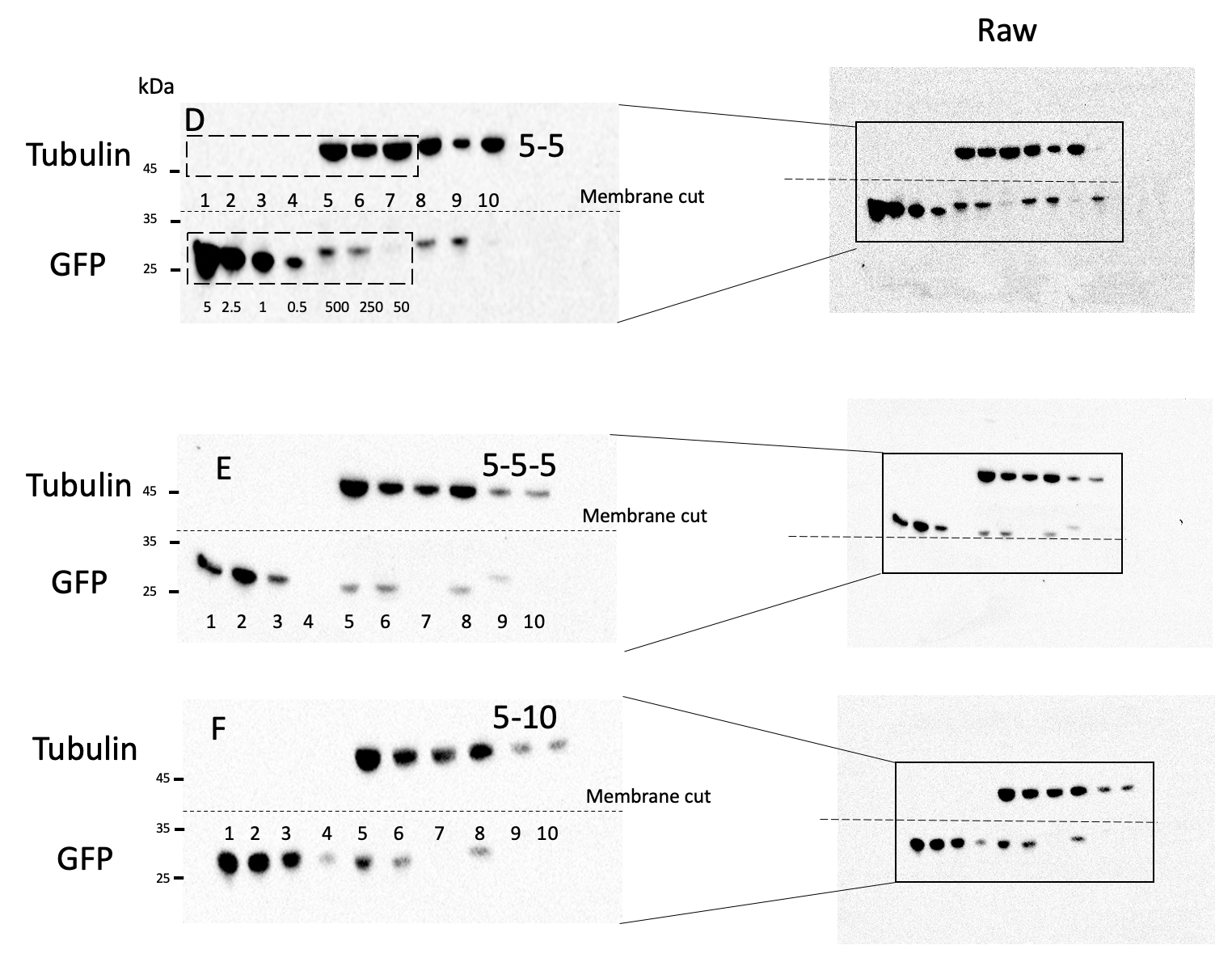


**Figure S1:** **(A-B-C)** HeLa In gel fluorescence assay acquired with an excitation wavelength of 501 nm for the 5-5, 5-5-5 and 5-10 delivery set-up. Lane 1); Ub-atto-488 10 μM; Lane 2); std Ub-atto-488 5 μM; Lane 3); std Ub-atto-488 1 μM; Lane 4); std Ub-atto-488 0.5 μM; Lane 5); control HeLa; lysate 6); lysate HeLa cells treated with 500μM external protein concentration; Lane 7); lysate HeLa cells treated with 250μM external protein concentration Lane 8); lysate HeLa cells treated with 50μM external protein concentration Lane 9); replicate lane 6; Lane 10); replicate lane 7; Lane 11); replicate lane 8. For all gel, the raw PAGEs are shown on the left. **(D-E-F)** HeLa Western blot assay against GFP (~ 27 kDa) and tubulin (~ 50 kDa) for the 5-5, 5-5-5 and 5-10 delivery set-up. Lane 1); GFP 5 μM; Lane 2); GFP 2.5 μM; Lane 3); GFP 1 μM; Lane 4); GFP 0.5 μM; Lane 5); control HeLa lysate 6); lysate HeLa cells treated with 500μM external protein concentration; Lane 7); lysate HeLa cells treated with 250μM external protein concentration Lane 8); lysate HeLa cells treated with 50μM external protein concentration Lane 9); replicate lane 6; Lane 10); replicate lane 7; Lane 11); replicate lane 8. For all Western blot, the raw acquisitions are shown on the right. The section of the gels and western blot reported in the main text are labelled in the same manner as in the manuscript main text in the relative gel and WB.


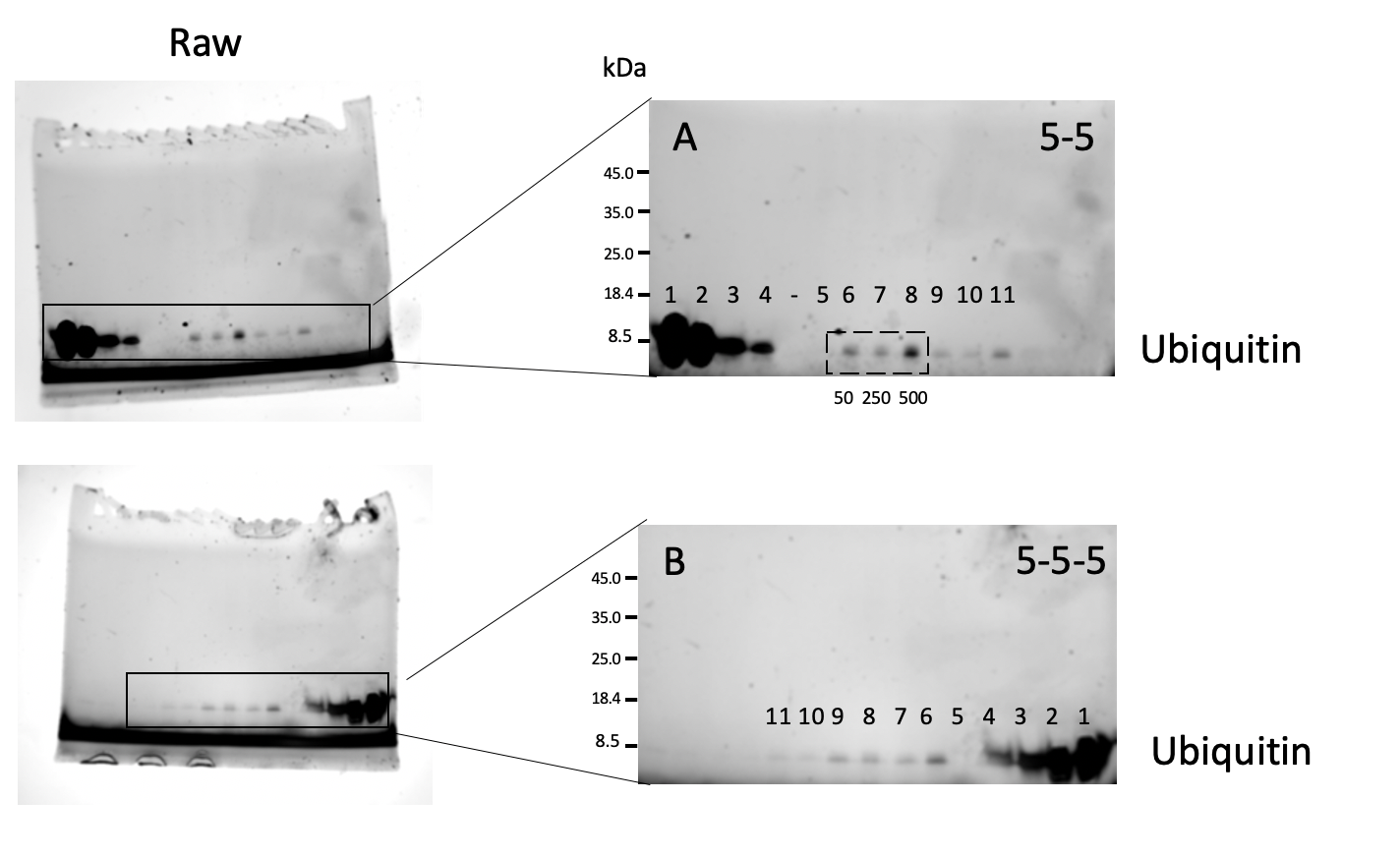


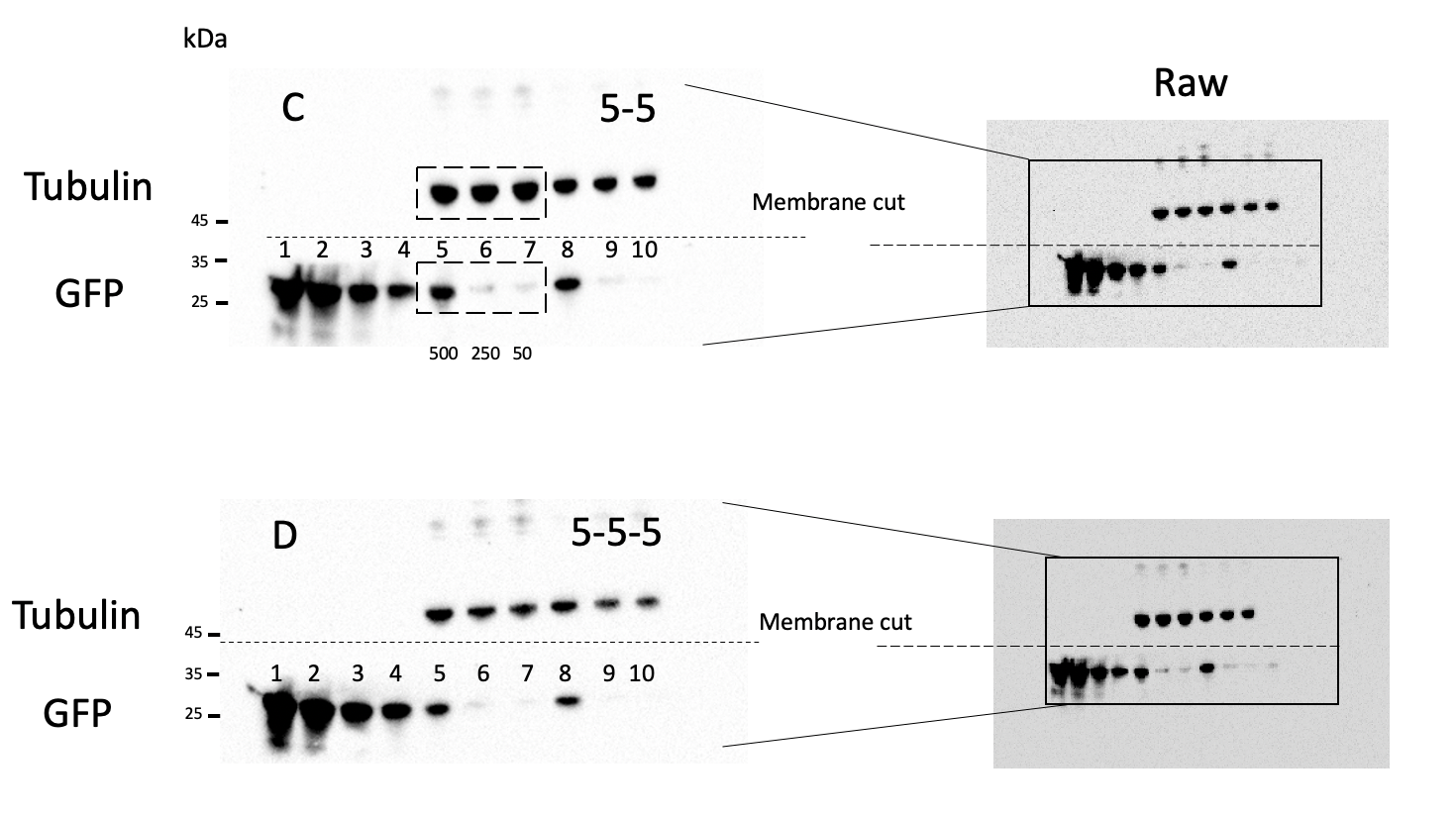


**Figure S2:** **(A-B-C)** Jurkat T In gel fluorescence assay acquired with an excitation wavelength of 501 nm for the 5-5, 5-5-5 and 5-10 delivery set-up. Lane 1); Ub-atto-488 10 μM; Lane 2); std Ub-atto-488 5 μM; Lane 3); std Ub-atto-488 1 μM; Lane 4); std Ub-atto-488 0.5 μM; Lane 5); control Jurkat T lysate 6); lysate Jurkat T cells treated with 500μM external protein concentration; Lane 7); lysate Jurkat T cells treated with 250μM external protein concentration Lane 8); lysate Jurkat T cells treated with 50μM external protein concentration Lane 9); replicate lane 6; Lane 10); replicate lane 7; Lane 11); replicate lane 8. For all gel, the raw PAGEs are shown on the left. **(D-E-F )** Jurkat T Western blot assay against GFP (~ 27 kDa) and tubulin (~ 50 kDa) for the 5-5, 5-5-5 and 5-10 delivery set-up. Lane 1); GFP 5 μM; Lane 2); GFP 2.5 μM; Lane 3); GFP 1 μM; Lane 4); GFP 0.5 μM; Lane 5); control Jurkat T lysate 6); lysate Jurkat T cells treated with 500μM external protein concentration; Lane 7); lysate Jurkat T cells treated with 250μM external protein concentration Lane 8); lysate Jurkat T cells treated with 50μM external protein concentration Lane 9); replicate lane 6; Lane 10); replicate lane 7; Lane 11); replicate lane 8. For all Western blot, the raw acquisitions are shown on the right. The cropped part of the gels and western blot reported in the main text are labelled in the same manner as in the manuscript main text in the relative gel and WB.


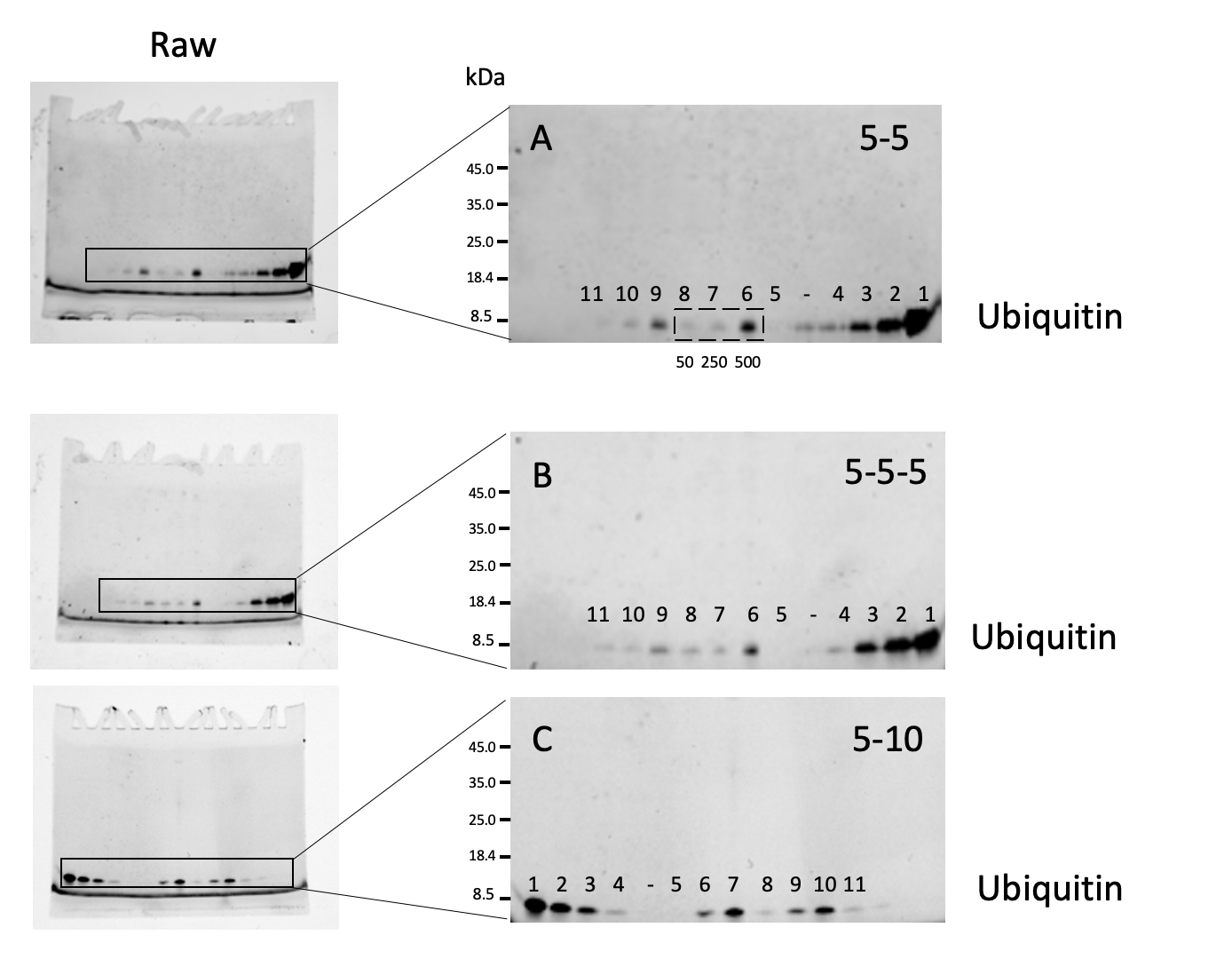


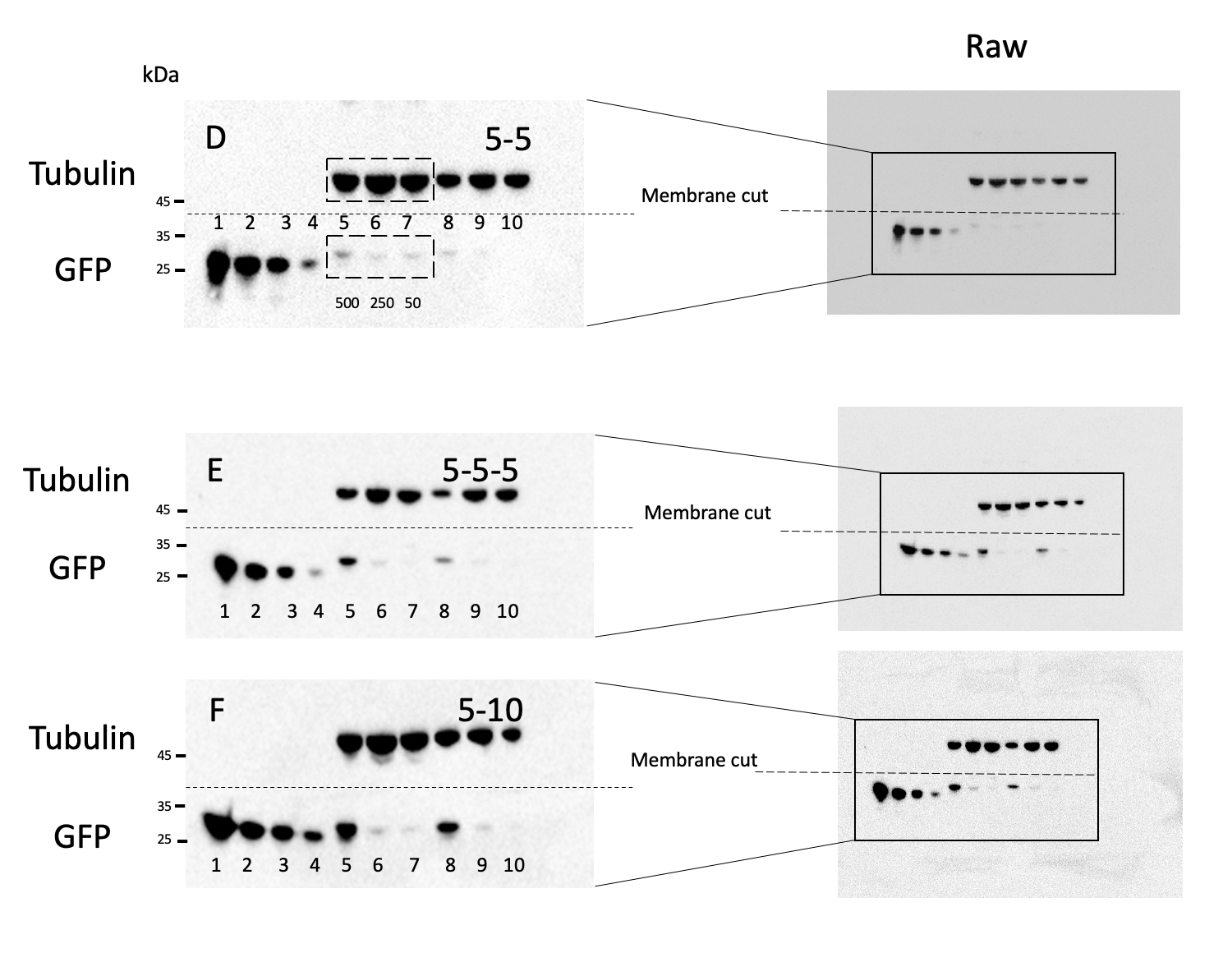


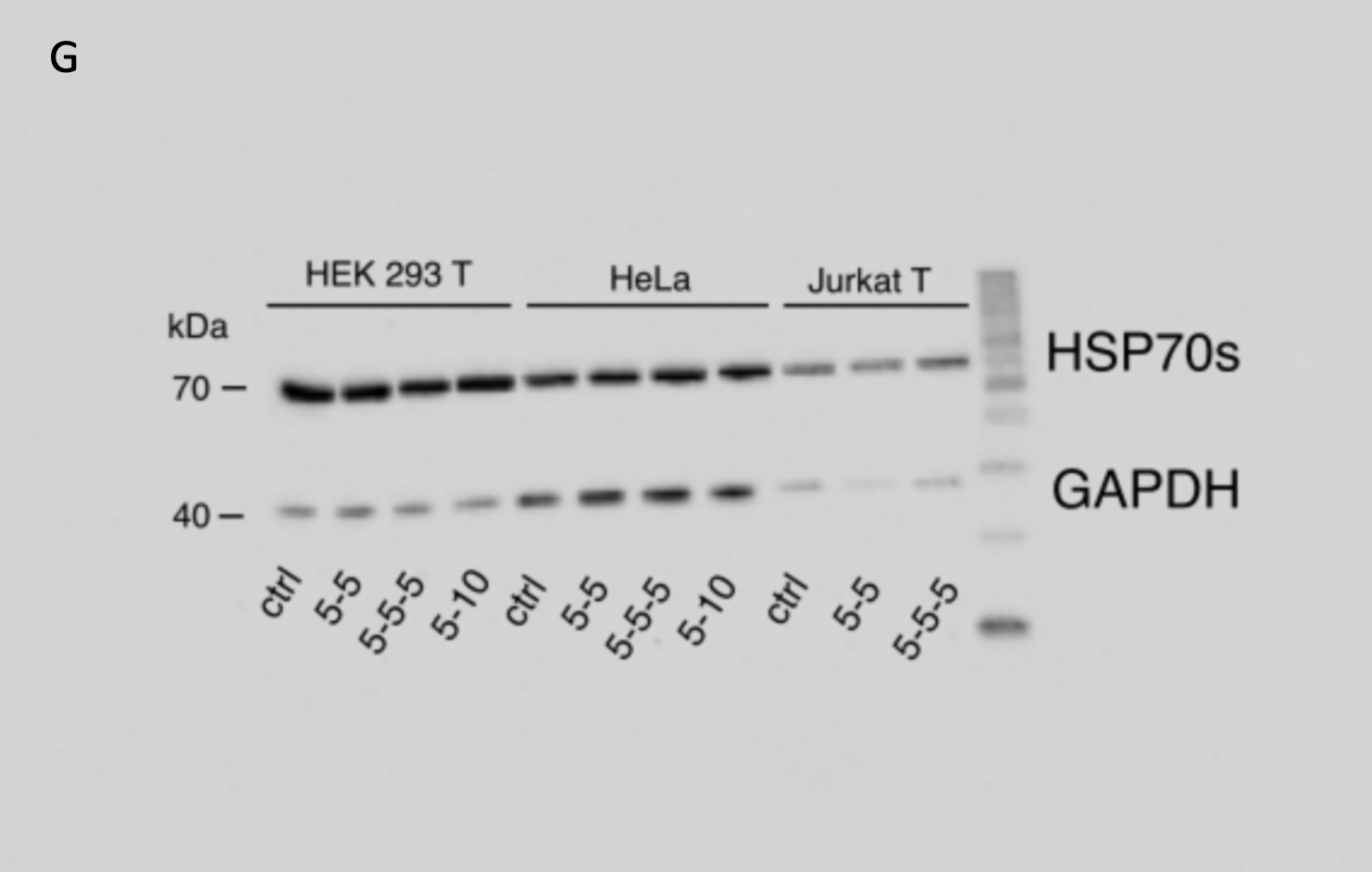


**Figure S3:** **(A-B-C)** HEK 293 T In gel fluorescence assay acquired with an excitation wavelength of 501 nm for the 5-5, 5-5-5 and 5-10 delivery set-up. Lane 1); Ub-atto-488 10 μM; Lane 2); std Ub-atto-488 5 μM; Lane 3); std Ub-atto-488 1 μM; Lane 4); std Ub-atto-488 0.5 μM; Lane 5); control HEK 293 T lysate 6); lysate HEK 293 T cells treated with 500μM external protein concentration; Lane 7); lysate HEK 293 T cells treated with 250μM external protein concentration Lane 8); lysate HEK 293 T cells treated with 50μM external protein concentration; Lane 9); replicate lane 6; Lane 10); replicate lane 7; Lane 11); replicate lane 8. For all gel, the raw PAGEs are shown on the left**. (D-E-F)** HEK 293 T Western blot assay against GFP (~ 27 kDa) and tubulin (~ 50 kDa) for the 5-5, 5-5-5 and 5-10 delivery set-up. Lane 1); GFP 5 μM; Lane 2); GFP 2.5 μM; Lane 3); GFP 1 μM; Lane 4); GFP 0.5 μM; Lane 5); control HEK 293 T lysate 6); lysate HEK 293 T cells treated with 500μM external protein concentration; Lane 7); lysate HEK 293 T cells treated with 250μM external protein concentration Lane 8); lysate HEK 293 T cells treated with 50μM external protein concentration Lane 9); replicate lane 6; Lane 10); replicate lane 7; Lane 11); replicate lane 8. For all Western blot, the raw acquisitions are shown on the right. The section of the gels and western blot reported in the main text are shown here by the red line. The cropped part of the gels and western blot reported in the main text are labelled in the same manner as in the manuscript main text in the relative gel and WB. (G) Raw western blot analysis described in main text Figure 1. The first row from the top represent the HSP70s expression levels while, the second one the GAPDH housekeeping protein normalization for all used cell lines (See main text Figure 1B for details).

**3D Z-slice confocal reconstruction
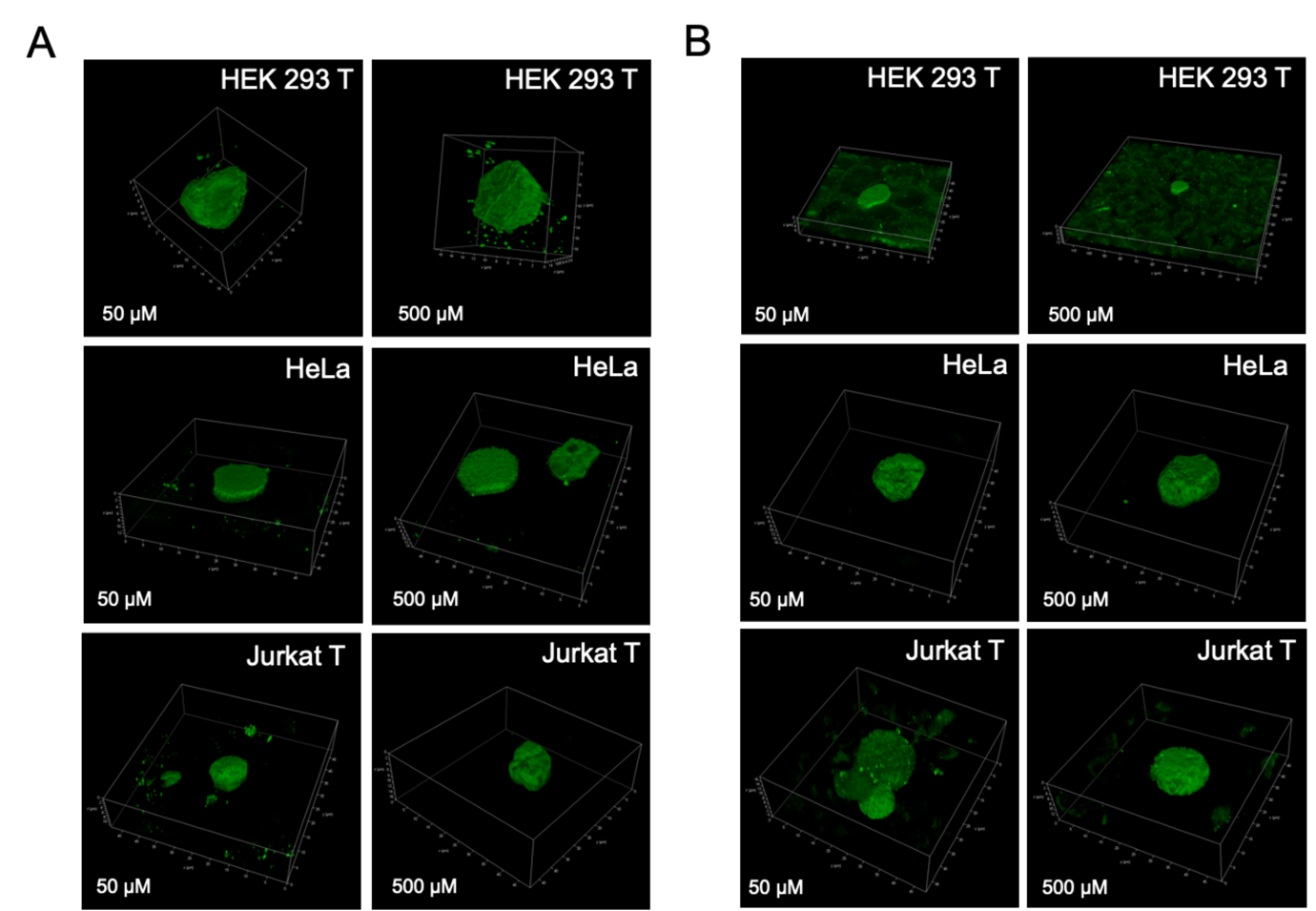
**

**Figure S4:** 3D confocal acquisitions of Ub-atto-488 (A) and GFP (B) HEK293T, HeLa, and Jurkat cell lines delivered with an external concentration of 50 µM and 500 µM. The 3D confocal images gave a clear view on the intactness of the cells. The round shape of the HeLa and HEK 293 T is mainly due to the fact that those cells images were acquired with detached cells. This is also confirmed by the comparison with the Jurkat T cells, which are cells that grow in suspension.

# **Ub-A28C and Ub-S20C/G35C ma-Px X-band cw-EPR in vitro spectra**


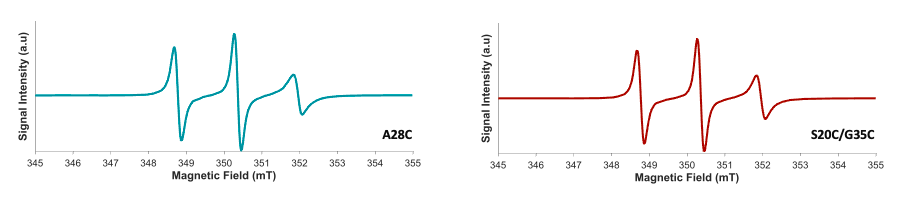


**Figure S5:** In vitro cw-EPR spctra acquired for spin labelled Ubiquitin A28C (left) and doubly labelled S20C/G35C (left). The spectra were recorded at a Protein spin concetration of 50 μM.

# **X-band Ub-A28C and Ub-S20C/G35C ma-Px delivery test**


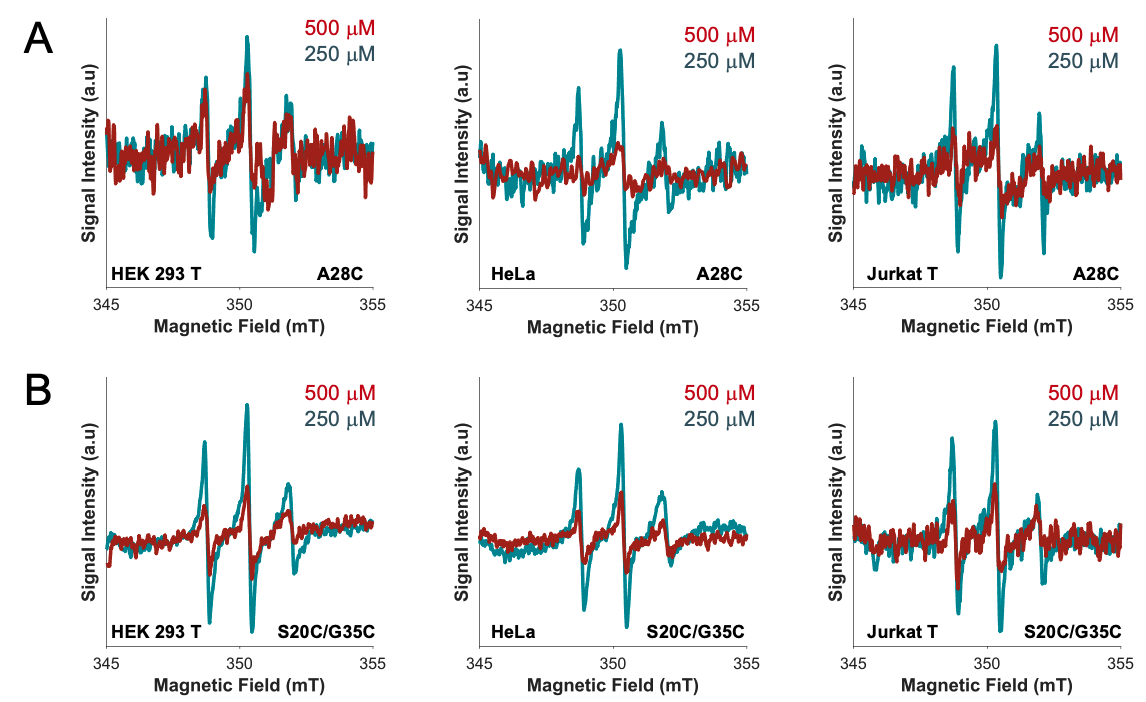


**Figure S6:** In cell spectra acquired for HEK 293 T, HeLa and Jurkat T delivered cells. (A) cw-EPR RT X-band spectra acquired for the three-cell line delivered with Ubiquitin-A28C labelled with ma-Px at two different external protein concentration. All samples were prepared using the 5-5 delivery set-up. (B) cw-EPR RT X-band spectra acquired for the three cell line delivered with Ubiquitin-S20C/G35C doubly labelled with ma-Px at two different external protein concentration. All samples were prepared using the 5-5 delivery set-up. An estimation of the bulk concentrations of the delivered protein samples were derived comparing the DI of the spectra with a curve made of ma-Px samples at known concentration.

# **In vitro Q-band EDFS Ub-S20C/G35C ma-Px**

#


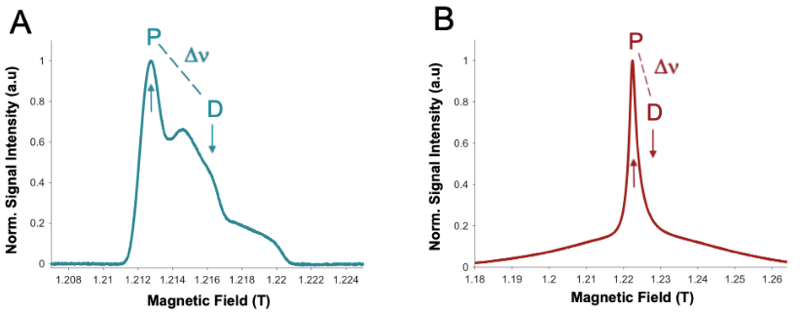


**Figure S7**: In vitro EDFS for 60 μM doubly labelled ma-Px Ubiquitin S20C/G35C (A) and 140 μM doubly labelled ma-Gd DOTA Ubiquitin S20C/G35C (B). The P and D symbol show the relative position of pump and detection frequency for the DEER experiments. The separation in frequency was kept in both cases at 70 MHz.

# **X-band cw-EPR in cell control experiments**


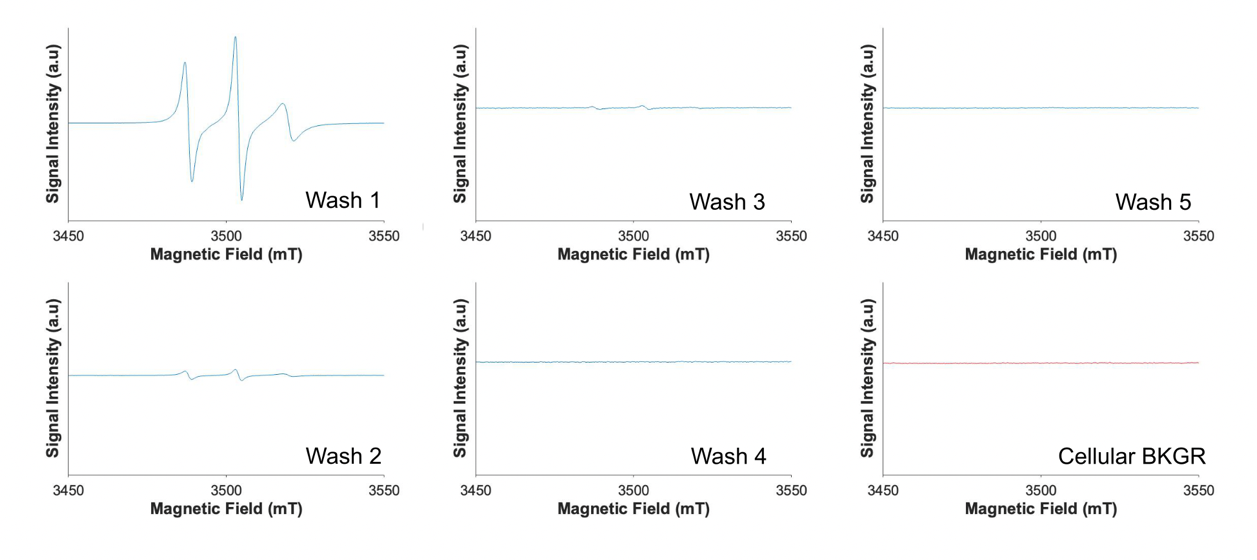


**Figure S8:** cw-EPR experiments acquired for the wash conducted after the protein delivery. (red spectra) Background acquired for the HEK cells at room temperature. All experiments were acquired with the same spectroscopical setup used for the in-cell measurements.

# **Q-band EDFS Ub-S20C/G35C maleimide-Gd-DOTA delivery test**


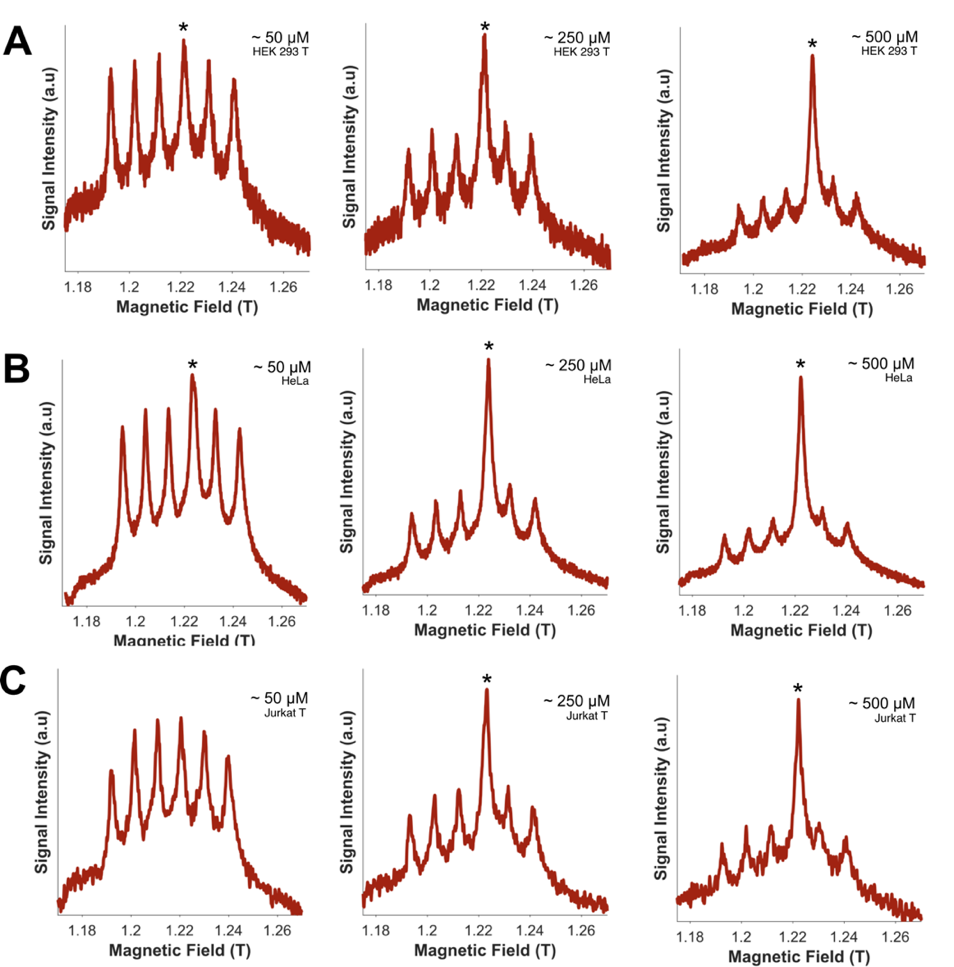


**Figure S9:** EDFS recorded for the in-cell samples delivered at three different external protein concentrations of Ub doubly labelled with the Gd3+ maleimide DOTA label. (A) HEK 293 T samples delivered with 50 mM, 250 mM and 500 mM external protein concentration. (B) HeLa samples delivered with 50 mM, 250 mM and 500 mM external protein concentration. (C) Jurkat T samples delivered with 50 mM, 250 mM and 500 mM external protein concentration. The representative Gadolinium signal arising from the labelled Ubiquitin is indicated by the *.

# **In cell Q-band EDFS Ub-S20C/G35C ma-Px**

#
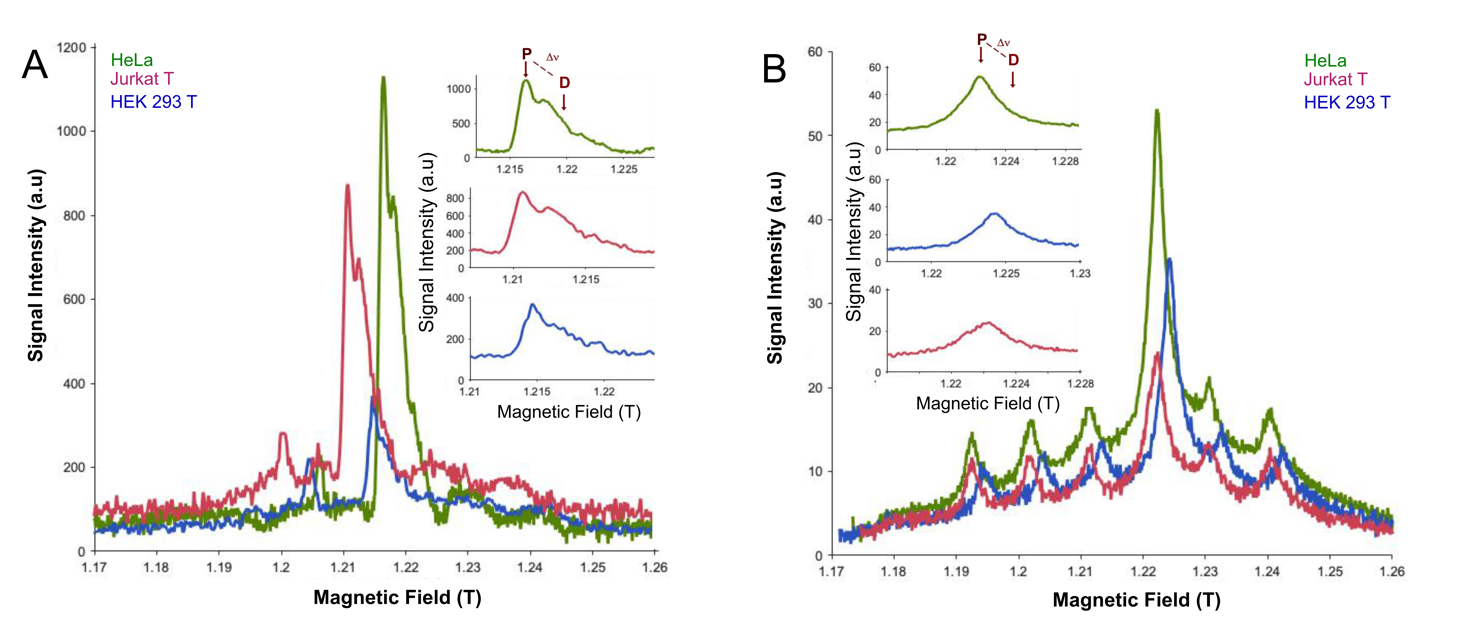


**Figure S10:** (A) In cell EDFS acquired for the different mammalian cell lines delivered with the doubly nitroxide labelled Ubiquitin S20C/G35C at 50K. All samples were delivered using the 5-5 set-up. (A-inset) Magnification of the nitroxide spectra region and graphical indication of the pump (P) and detection (D) relative positions used for the DEER experiments. (B) In cell EDFS acquired for the different mammalian cell lines delivered with the doubly Gadolinium labelled Ubiquitin S20C/G35C at 10K. All samples were delivered using the 5-5 set-up. (B-inset) Magnification of the Gadolinium spectra region and graphical indication of the pump (P) and detection (D) relative positions used for the DEER experiments.

# **In vitro Nitroxide Gadolinium DEER/PELDOR set-up and data analysis**


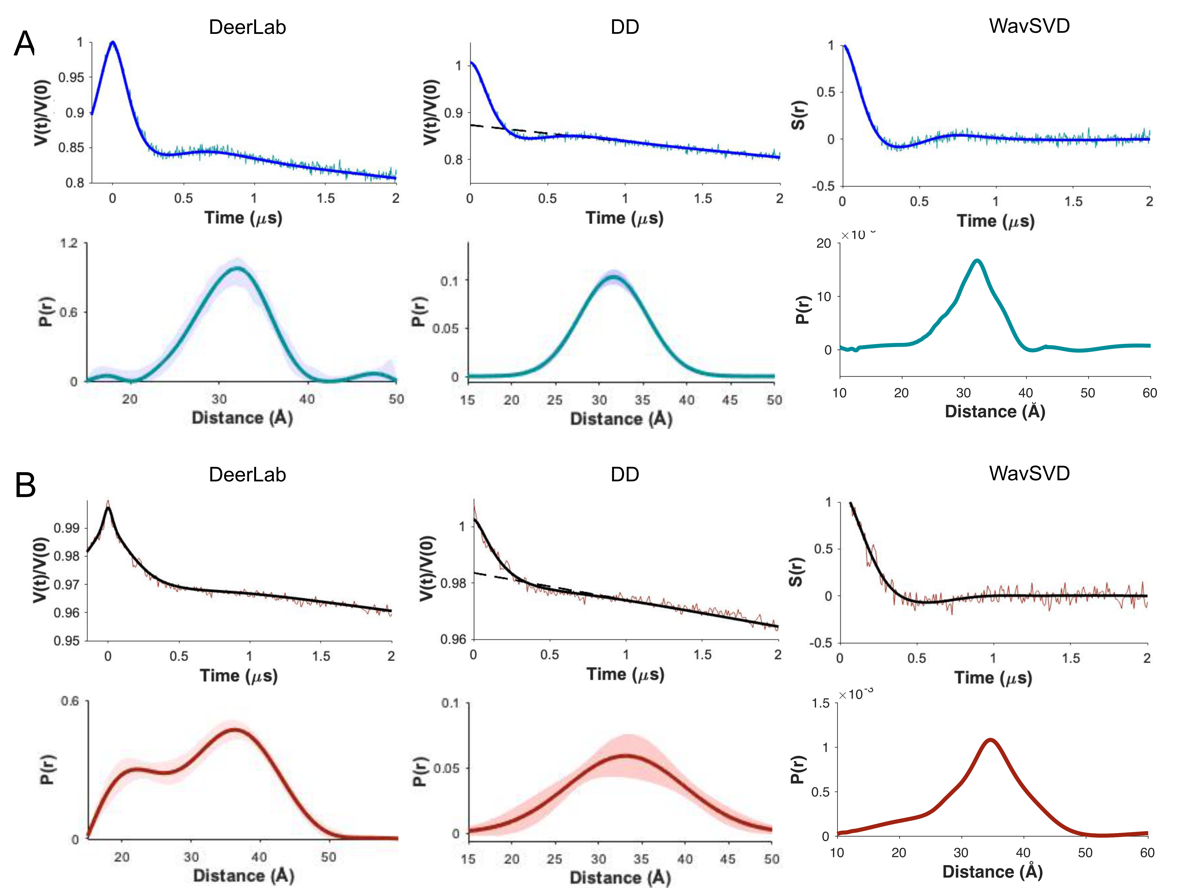


**Figure S11:** In vitro DEER trace analysis conducted using three different suited programs. (A) DEER trace and relative extracted distance distribution derived from DeerLab,DD and WavSVD programs doubly labelled maleimide-Proxyl Ubiquitin sample. (B) DEER trace and relative extracted distance distribution derived from DeerLab,DD and WavSVD programs for the doubly labelled Gd-DOTA-maleimide Ubiquitin sample. In both cases in the WavSVD trace plot, we reported the experimental data and the relative reconstitute trace. The detailed description of the acquisition parameters used are shown in Table.1.

# **In cell Nitroxide DEER/PELDOR set-up and data analysis**


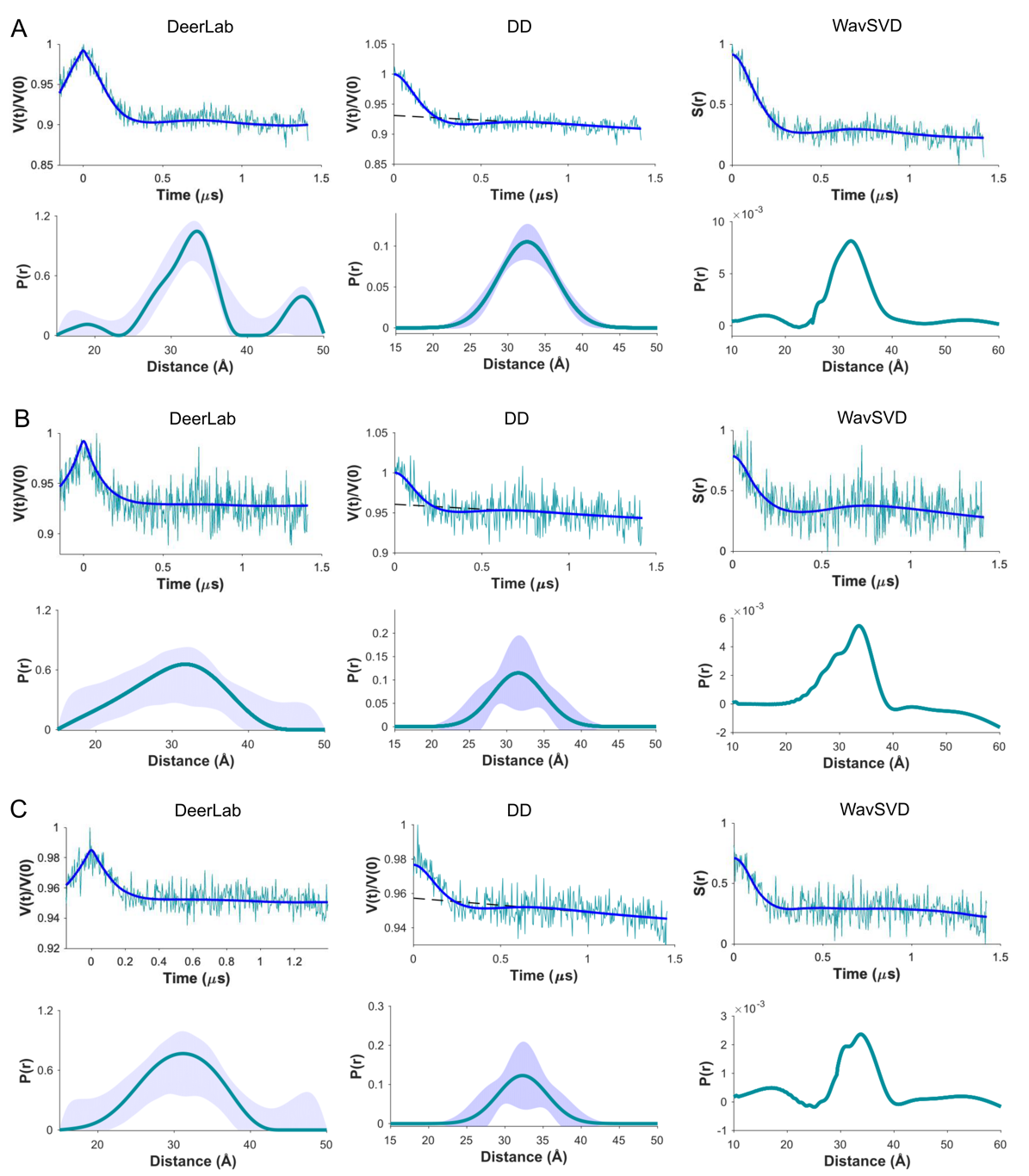


**Figure S12:** (A) DEER trace and relative extracted distance distribution derived from DeerLab, DD and WavSVD programs for the doubly labelled maleimide-Proxyl Ubiquitin delivered HeLa cell. (B) DEER trace and relative extracted distance distribution derived from DeerLab, DD and WavSVD programs for the doubly labelled maleimide-Proxyl Ubiquitin delivered HEK 293 T cell. (C) DEER trace and relative extracted distance distribution derived from DeerLab, DD and WavSVD programs for the doubly labelled maleimide-Proxyl Ubiquitin delivered Jurkat T cell. In all cases in the WavSVD trace plot, we reported the experimental data and the relative SVD reconstitute trace. The detailed description of the acquisition parameters used are shown in Table.1.

# **In cell Gadolinium DEER/PELDOR set-up and data analysis**

# **
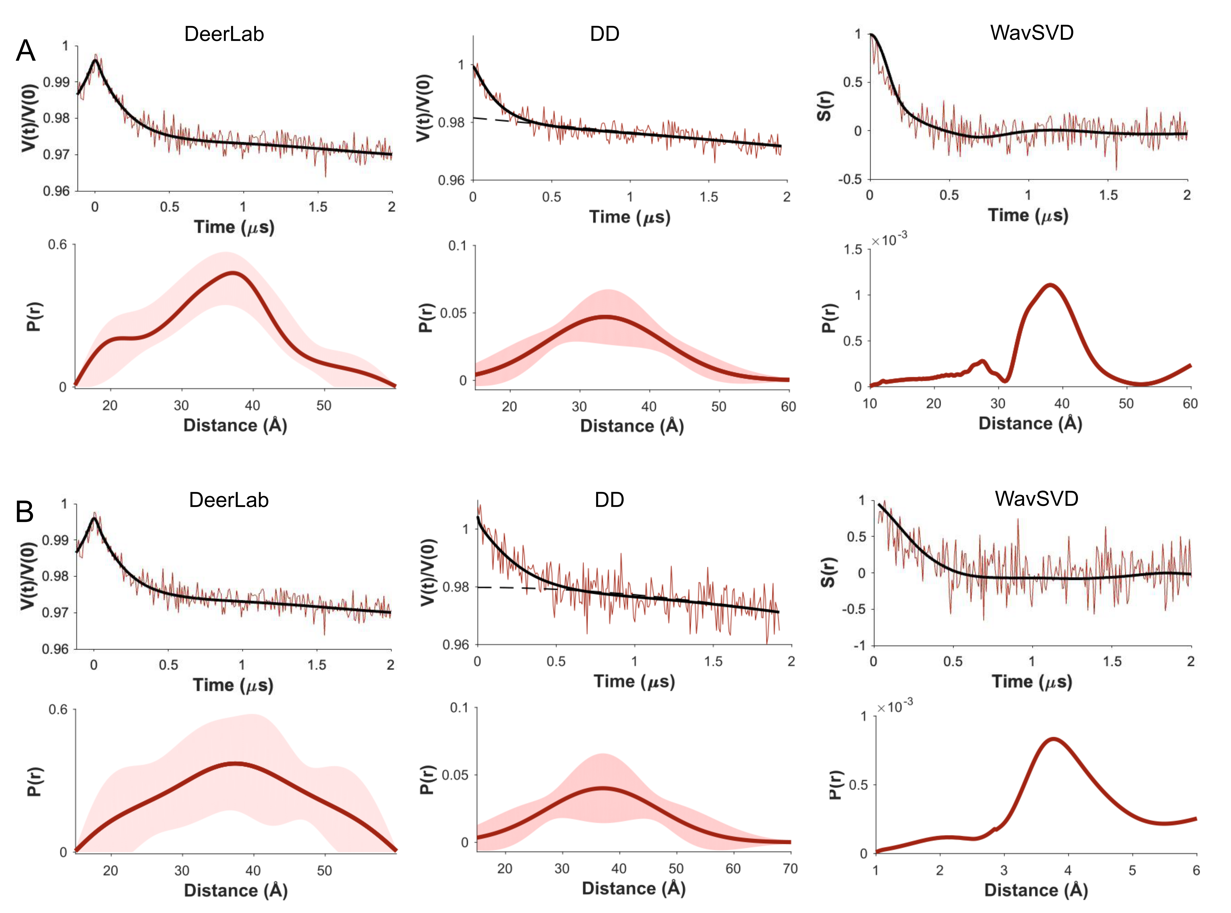
**

**Figure S13:** (A) DEER trace and relative extracted distance distribution derived from DeerLab, DD and WavSVD programs for the doubly labelled maleimide-Gd-DOTA Ubiquitin S20C/G35C delivered HeLa cell. (B) DEER trace and relative extracted distance distribution derived from DeerLab, DD and WavSVD programs for the doubly labelled maleimide-Gd-DOTA Ubiquitin S20C/G35C delivered HEK 293 T cell. In all cases in the WavSVD trace plot, we reported the experimental data and the relative SVD reconstitute trace. The detailed description of the acquisition parameters used are shown in Supplementary Table.1.

# **In cell Q-band EDFS Ub-S20C/G35C Ma-DOTA-Gd^3+^.**


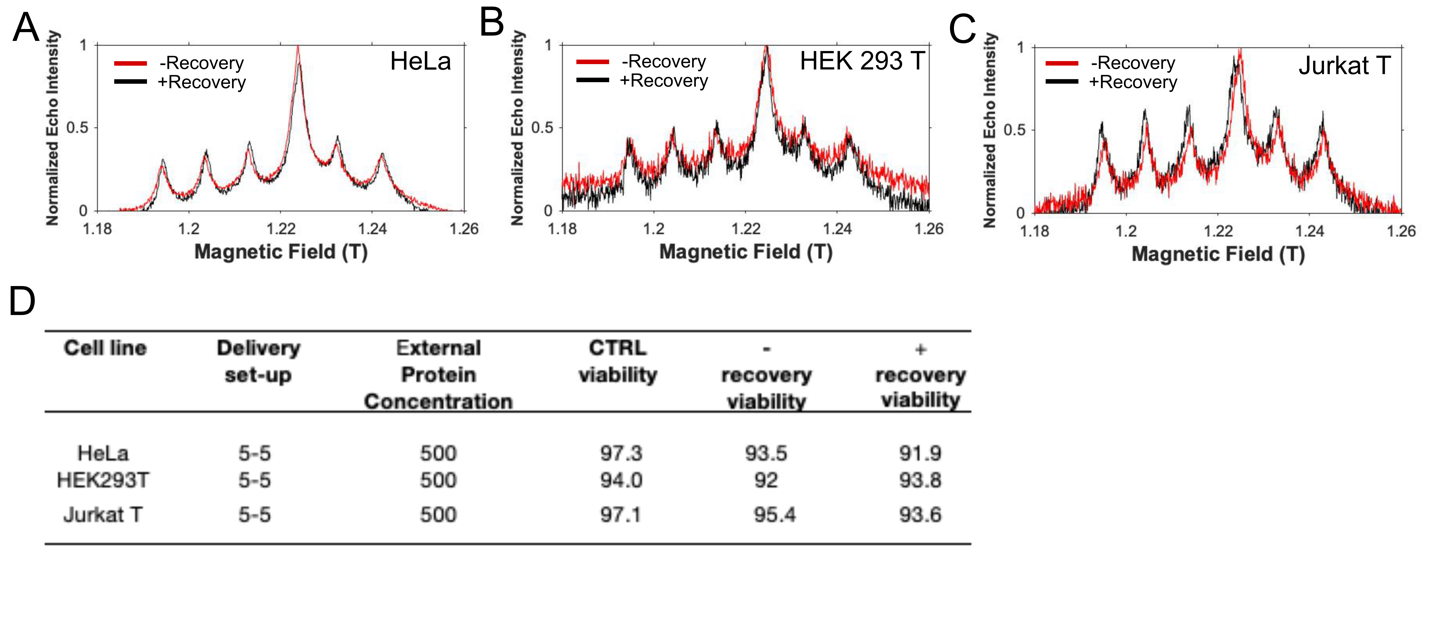


**Figure S14:** Recovery test conducted on the delivered HeLa (A), HEK 293 T (B) and Jurkat T (C) cells delivered with the doubly labelled Ubiquitin S20C/G35C Ma-DOTA-Gd3+. All ESE spectra were acquired optimizing the spectroscopical set-up as reported in the method section. For all cell lines the red spectra have been acquired right after the delivery and the preparation time without using any time of recovery. On the other hand, the black spectra were acquired after a recovery time in the complete medium for a period ranging in between 50-90 minutes (at 37 °C cells were placed in the EPR tube and the frozen in liquid nitrogen for the measurements. The Trypan blue viability value and the delivery set up are reported in the table D.

# **In cell Q-band Hahn echo decay curves**

#
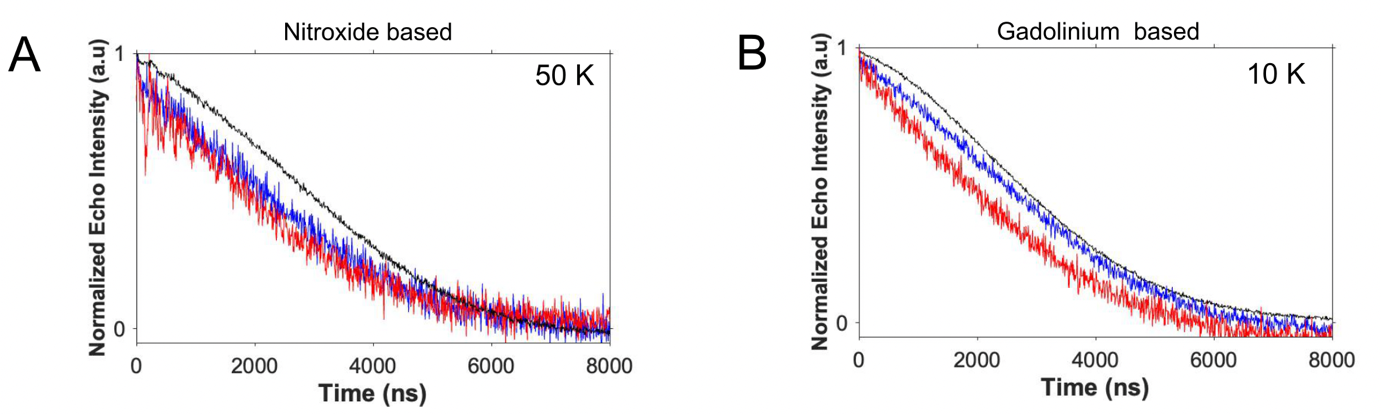


**Figure S15:** Hahn Echo decay curves acquired for the in cell delivered HeLa (red spectrum) and HEK293T (blue spectrum) cells with the nitroxide doubly labelled Ubiquitin S20C/G35C (A) and doubly labeled with the gadolinium DOTA maleimide (B). The nitroxide experiments were acquired at 50 K, while the gadolinium based where acquired at 10 K. The Hanh echo experiments were optimized for each experiment using an increment delay of 4 ns. The in vitro sample was at a concentration of 60 μM for the doubly nitroxide labelled and 140 μM for the doubly maleimide- gadolinium DOTA one.

# **Table. 1:** Parameters used for the in vitro and in cell DEER experiments on the doubly labelled nitroxide and gadolinium Ubiquitin based samples

|  | in vitro  Nx | in vitro  Gd | HEK  Nx | HeLa  Nx | Jurkat T  Nx | HEK  Gd | HeLa  Gd |
| --- | --- | --- | --- | --- | --- | --- | --- |
| π−Pump (ns) | 34 | 28* | 30 | 38 | 30 | 38* | 36* |
| π−Det. Pulse (ns) | 70 | 46* | 64 | 70 | 60 | 70* | 60* |
| Δν (MHz) | -70 | -70 | -70 | -70 | -70 | -70 | -70 |
| τ_1_ (ns) | 200 | 750 | 750 | 750 | 750 | 750 | 750 |
| τ_2_ (ns) | 2500 | 3000 | 2000 | 2000 | 2000 | 3000 | 3000 |
| Δτ (ns) | 4 | 8 | 4 | 4 | 4 | 8 | 8 |
| SRT(ms) | 2500 | 500 | 1500 | 1500 | 1500 | 500 | 500 |
| SPP | 1-50 | 1-50 | 1-50 | 1-50 | 1-50 | 1-50 | 1-50 |
| Temperture (K) | 50 | 10 | 50 | 50 | 50 | 10 | 10 |
| Phase Cycling | 8 | 8 | 8 | 8 | 8 | 8 | 8 |
| Cell number (x10^6^) | - | - | ~ 5.2 | ~ 5.7 | ~ 6.2 | ~ 6 | ~ 5.9 |
| Acquisition time (h) | 3h | 1h | ~ 18/20 | ~18/20 | ~ 18/20 | ~ 25 | ~ 25 |

*For the Gd based DEER Gaussian pulses were optimized instead of the rectangular one to reduce the 2+1 modulation effect.

# **Table. 2:** Nitroxide doubly labelled Ub S20C/G35C in-cell/in vitro derived distances

| Ub S20C/G35C ma-Px | DeerLab | DD | WavSVD |
| --- | --- | --- | --- |
| In vitro | 32 | 31 | 32 |
| HEK 293 T | 32 | 31 | 33 |
| HeLa | 33 | 32 | 32 |
| Jurkat T | 31 | 32 | 32 |

# **Table. 3:** Gadolinium doubly labelled Ub S20C/G35C in-cell/in vitro derived distances

| Ub S20C/G35C ma-DOTA-Gd^3+^ | DeerLab | DD | WavSVD |
| --- | --- | --- | --- |
| In vitro | 36 | 33 | 34 |
| HEK 293 T | 37 | 36.7 | 37 |
| HeLa | 37 | 34 | 37 |
